# Astrocytic CXCL5 hinders microglial phagocytosis of myelin debris and aggravates white matter injury in chronic cerebral ischemia

**DOI:** 10.1186/s12974-023-02780-3

**Published:** 2023-05-03

**Authors:** Qian Cao, Jian Chen, Zhi Zhang, Shu Shu, Yi Qian, Lixuan Yang, Lushan Xu, Yuxin Zhang, Xinyu Bao, Shengnan Xia, Haiyan Yang, Yun Xu, Shuwei Qiu

**Affiliations:** 1 Department of Neurology, Drum Tower Hospital, Medical School and The State Key Laboratory of Pharmaceutical Biotechnology, Institute of Translational Medicine for Brain Critical Diseases, Nanjing University, Nanjing, 210008 China; 2 Department of Neurology, Nanjing Drum Tower Hospital Clinical College of Traditional Chinese and Western Medicine, Nanjing University of Chinese Medicine, Nanjing, 210008 China; 3 Jiangsu Key Laboratory for Molecular Medicine, Medical School of Nanjing University, Nanjing, 210008 China; 4 Jiangsu Provincial Key Discipline of Neurology, Nanjing, 210008 China

**Keywords:** CXCL5, BCAS, Astrocyte, Microglia, Phagocytosis

## Abstract

**Background:**

Chronic cerebral ischemia induces white matter injury (WMI) contributing to cognitive decline. Both astrocytes and microglia play vital roles in the demyelination and remyelination processes, but the underlying mechanism remains unclear. This study aimed to explore the influence of the chemokine CXCL5 on WMI and cognitive decline in chronic cerebral ischemia and the underlying mechanism.

**Methods:**

Bilateral carotid artery stenosis (BCAS) model was constructed to mimic chronic cerebral ischemia in 7–10 weeks old male mice. Astrocytic *Cxcl5* conditional knockout (cKO) mice were constructed and mice with *Cxcl5* overexpressing in astrocytes were generated by stereotactic injection of adeno-associated virus (AAV). WMI was evaluated by magnetic resonance imaging (MRI), electron microscopy, histological staining and western blotting. Cognitive function was examined by a series of neurobehavioral tests. The proliferation and differentiation of oligodendrocyte progenitor cells (OPCs), phagocytosis of microglia were analyzed via immunofluorescence staining, western blotting or flow cytometry.

**Results:**

CXCL5 was significantly elevated in the corpus callosum (CC) and serum in BCAS model, mainly expressed in astrocytes, and *Cxcl5* cKO mice displayed improved WMI and cognitive performance. Recombinant CXCL5 (rCXCL5) had no direct effect on the proliferation and differentiation of OPCs in vitro. Astrocytic specific *Cxcl5* overexpression aggravated WMI and cognitive decline induced by chronic cerebral ischemia, while microglia depletion counteracted this effect. Recombinant CXCL5 remarkably hindered microglial phagocytosis of myelin debris, which was rescued by inhibition of CXCL5 receptor C-X-C motif chemokine receptor 2 (CXCR2).

**Conclusion:**

Our study revealed that astrocyte-derived CXCL5 aggravated WMI and cognitive decline by inhibiting microglial phagocytosis of myelin debris, suggesting a novel astrocyte-microglia circuit mediated by CXCL5-CXCR2 signaling in chronic cerebral ischemia.

**Supplementary Information:**

The online version contains supplementary material available at 10.1186/s12974-023-02780-3.

## Background

White matter injury (WMI) is the most important feature of cerebral small vascular disease (CSVD), which is closely associated with the cognitive decline and serves as a prime therapeutic target for alleviating cognitive decline [1]. Currently, there is no effective therapeutic stratagem for alleviating the WMI-associated cognitive decline in CSVD due to the unclear mechanism underlying demyelination and remyelination. A better understanding of WMI pathogenesis could favor developing therapies for the prevention and/or treatment of CSVD.

Microglia and astrocytes play important roles in regulating the demyelination and remyelination process in CSVD. Therefore, controlling the microglia- and astrocyte-mediated chronic neuroinflammation is considered as an efficient therapeutic strategy for WMI [2]. For example, cornel iridoid glycoside reduced microglia-mediated inflammatory response via the NOD-like receptor thermal protein domain associated protein 3 (NLRP3)/calpain pathway and attenuated myelin loss and cognitive decline in a rat model of chronic cerebral ischemia [3]. In addition, both microglia and astrocytes could also promote remyelination via releasing neurotrophic factors, like insulin-like growth factor l (IGF-1) [4, 5]. Accumulated myelin debris was proven to hinder white matter repair through directly inhibiting remyelination or promoting secondary inflammatory cascade [6]. Interestingly, both microglia and astrocytes were reported to manifest capacity in clearing myelin debris and promoting white matter repair. For example, Zheng et al. reported that A1 astrocytes inhibited microglial phagocytosis of myelin debris via an astrocytic C3-microglial C3aR axis, aggravating WMI after intracerebral hemorrhage [7]. Our previous study reported that pentoxifylline (PTX) alleviated ischemic WMI in BCAS model via facilitating microglial phagocytosis of myelin debris [8]. Thus, key molecules modulating glial cell function may be primary therapeutic targets for WHI in CSVD.

CXCL5, also named epithelial neutrophil-activating peptide-78 (ENP-78) or lipopolysaccharide-induced CXC chemokine (LIX), is a small cytokine belonging to CXC chemokine family. It is expressed in various cells, including glial cells and epithelial cells [9]. CXCL5 was reported to be significantly elevated in response to injury in central nervous system (CNS) and participate in neuro-inflammatory response and WMI. For example, Wang et al. found that CXCL5 was remarkably increased in the immature brain after LPS-sensitized hypoxic ischemia, mainly in microglia and endothelial cells, and inhibition of CXCL5 with a selective nonpeptide inhibitor of its receptor CXCR2 could attenuate microglial activation, blood brain and barrier (BBB) damage, astrogliosis and promote myelination [10]. Conversely, Liu et al. reported that CXCL5 and CXCR2 were increased after optic nerve and lens injury, and that recombinant CXCL5 (rCXCL5) could promote microglial activation, retinal ganglion cell (RGC) survival and axonal regeneration [11]. Here, using transcriptome sequencing analysis, we found that CXCL5 was remarkably up-regulated in the brain of mice with chronic cerebral ischemia, mainly in astrocytes. Currently, the role of CXCL5, especially astrocytic CXCL5, in CSVD remains unclear, and we hypothesize that astrocytic CXCL5 exacerbates WMI and cognitive decline associated with chronic cerebral ischemia. Here, we further investigated the impact of CXCL5 on WMI and related cognitive decline and explored the potential mechanisms.

## Materials and methods

### Mice

C57BL/6J male mice (7–10 weeks old, weighing 25–30 g), astrocyte-specific *Cxcl5* knockout mice (*Cxcl5*^fl/fl^; GFAP-Cre) and age-matched wild-type littermates (*Cxcl5*^fl/fl^) were purchased from the Model Animal Research Center of Nanjing University (China), with the construction strategy diagram shown in Additional file 1: Fig. S1. Primer sequences for identification of genetic mice are shown in Additional file 1: Table S1. All animals were maintained under specific pathogen-free conditions and housed at a controlled temperature of 22–24 °C and humidity of 50–60% for 12 h cycle alternating light/dark, with standard laboratory food and water at will. All animal experiments were performed under the guidance of the Animal Care Committee of Nanjing University (approval number: 2021AE01074).

### Cell culture

Primary glial cell cultures were isolated from 1-day-old C57BL/6J mice or astrocyte specific *Cxcl5* knockout mice as previously described [12, 13]. Briefly, the brain membrane was removed and the cerebral cortex (CTX) was digested with 0.25% tripsin EDTA for 10 min. The Dulbecco’s Modified Eagle Medium (DMEM, Invitrogen, USA) containing 10% fetal bovine serum (FBS, Hyclone, USA) was then added to terminate the digestion. The cells were centrifuged at 800 rpm at 37 °C for 10 min. The supernatant was aspirated and the cells were cultured in 75 cm^2^ flasks for 10–13 days in DMEM, which was supplemented with 10% FBS and 100 U/ml antibiotics. Microglia and astrocytes were isolated by shaking for subsequent tests. OPCs were isolated from brain of mice at postnatal day 1 and cultured on poly-D-Lysine (Sigma, USA) -coated plates in OPC proliferation medium containing DMEM/F12, 2% B27, 100 U/ml antibiotics, 30 ng/ml PDGF-AA (GenScript, China) and 10 ng/ml bFGF (GenScript). After 5–7 days, cells were switched to differentiation medium containing DMEM/F12, B27, antibiotics, 50 ng/ml T3 (Triiodothyronine, R&D Systems, USA) and 20 ng/ml CNTF (GenScript). Primary cortical neurons were prepared from fetal mice brains and cultured in medium containing neurobasal, 2% B27, and 25 nM glutamine as previously described [14].

### BCAS surgery

As previously described [15], BCAS surgeries were performed on male mice aged approximately 7–10 weeks old. Mice were anesthetized with avertin (2.5%, Sigma) and placed in the supine position. The common carotid artery was carefully exposed through a midline incision. A microcoil (inner diameter 0.18 mm, pitch 0.50 mm, total length 2.5 mm, purchased from Sawane Spring Co.) was wrapped around the common carotid artery to induce chronic cerebral hypoperfusion. Thirty minutes later, the same procedure was performed on the other common carotid artery. Sham-operated mice underwent all procedures except microcoil implantation. A grand total of 204 mice (35 sham-operated and 169 BCAS mice) were used. The mortality during BCAS surgery was 2.0% (3/169). At 2 months after surgery, the mice were subjected to behavioral tests and magnetic resonance imaging (MRI), and then sacrificed for subsequent experiments.

### Transcriptome sequencing

Total RNA was extracted from corpus callosum (CC) tissues and quantified by the NanoDrop ND-2000 (Thermo Scientific). The RNA integrity was assessed using Agilent Bioanalyzer 2100 (Agilent Technologies). Next, total RNA were transcribed to double strand cDNA, then synthesized into cRNA and labeled with Cyanine-3-CTP. The labeled cRNAs were hybridized onto the microarray. After washing, the arrays were scanned by the Agilent Scanner G2505C (Agilent Technologies). Feature Extraction software (version 10.7.1.1, Agilent Technologies) was used to analyze array images to obtain raw data. Genespring (version 13.1, Agilent Technologies) was used to perform basic analysis on the raw data. Differentially expressed genes were then identified by fold change and P value calculated by t-test. Afterwards, GO analysis and KEGG analysis were then used to determine the roles of these differentially expressed mRNAs.

### Western blot

Cells and tissues were lysed in Radio Immunoprecipitation Assay (RIPA) buffer containing protease inhibitors. The extracted proteins were quantified using BCA kit (Thermo Fisher Scientific), denatured separated with SDS-PAGE and transferred to polyvinylidene fluoride (EMD Millipore) membrane. The membranes were blocked with 5% skim milk for 2 h at room temperature and incubated with primary antibody overnight at 4 °C. The next day, the membranes were immersed in secondary antibodies for 2 h. Targeted proteins were detected using the Tanon bioluminescence instrument and analyzed by ImageJ software (ImageJ 1.5, NIH, USA). Details of the antibodies are given in Additional file 1: Table S2.

### Real-time quantitative PCR

Total RNA was extracted from tissues or cells using TRIzol reagent (Invitrogen). The extracted RNA was reverse transcribed to cDNA using PrimeScript RT Reagent Kit (Vazyme, China). Quantitative real-time PCR (qPCR) was performed on an ABI 7500 PCR instrument (Applied BioSystems, USA) using the SYBR GREEN kit (Vazyme, China). The relative quantification of genes was analyzed by the 2^−ΔΔCt^ method and normalized with GAPDH values. Primer sequences are listed in Additional file 1: Table S3.

### Myelin debris preparation and stimulation

Myelin debris was prepared as previously described [16]. Briefly, the mice were euthanized and the brains were cut into pieces (approximately 5 mm^3^) in 0.32 M sucrose solution on ice. The solutions were then homogenized and transfered to the top of the 0.83 M sucrose solution, taking care not to mix the two layers. After centrifugation at 100,000 rpm for 45 min at 4 °C, the crude myelin debris was collected from the interface of the two sucrose densities, dissolved in Tris·Cl buffer solution and homogenized for 30–60 s. After two rounds of centrifugation, the pellets were resuspended in sterile phosphate buffer solution (PBS) and centrifuged at 22,000 rpm for 10 min at 4 °C. Finally, myelin debris was resuspended in PBS to a final concentration of 100 mg/ml. Primary microglia pretreated with the CXCR2 inhibitor SB225002 (10 nM) and rCXCL5 (25 μg/ml) were stimulated with myelin debris (0.01 mg/ml) for different times. Myelin debris was stained with DiD (a far-red plasma membrane fluorescent probe, Beyotime, China) or DiI (a red plasma membrane fluorescent probe, Beyotime, China) for flow cytometry or immunofluorescence.

### Electron microscopy

After the mice were sacrificed, the CC was removed and immersed in 2.5% glutaraldehyde. Sample preparation and image acquisition were performed by Servicebio (China). Images were screened by transmission electron microscopy (Hitachi, HT7800, Japan). The G-ratio (diameter of the axon/the diameter of the whole myelinated fiber) of at least 100 myelin sheaths per animal was calculated using ImageJ.

### Immunofluorescence staining

The mice were anesthetized, perfused with PBS and successively fixed in 4% paraformaldehyde. The brains were quickly removed and put into 4% paraformaldehyde. Then, the brains were dehydrated in 15% and 30% sucrose for 24 h each and cut into 20 μm coronal sections (anterior–posterior distance from Bregma: 1.0 mm to 0.2 mm) with a cryostat microtome (Leica, Wetzlar, Germany). First, the slices were permeabilized with 0.25% Triton X-100, then they were blocked in 2% bovine serum albumin (BSA) for 1–2 h. Afterwards, the slices were incubated primary antibodies overnight at 4 °C. Finally, the tissue sections were incubated with secondary antibody and stained with 4',6-diamidino-2-phenylindole (DAPI) in the dark condition. Images were acquired using with a confocal fluorescence microscope (Olympus FV3000, Japan) and analyzed with ImageJ software. For cells, the cells were fixed with 4% paraformaldehyde and then treated with 0.25% PBST, for permeabilization, and the subsequent procedures were similar to immunofluorescence staining of frozen sections.

### Flow cytometry

Primary microglia pretreated with CXCR2 inhibitor SB225002 (10 nM) and rCXCL5 (25 μg/ml) for 0.5 h were incubated with myelin fragments (0.01 mg/ml) stained with DiD’ solid for 1 h. After trypsinization, the cells were collected, centrifugated and resuspended with PBS. The proportion of microglia phagocytosing myelin debris (DiD^+^ cells/total cells) was analyzed by the flow cytometer (BD Accuri C6, USA).

### Astrocyte isolation

Astrocytes were isolated as described previously [17, 18]. Mice were sacrificed by decapitation, and the brains were immediately removed to isolate CTX and CC tissue. Brains were diced into 1 mm^3^ pieces using a scalpel blade. The tissues were incubated in an oxygenated papain solution containing 100 units of papain (Worthington Biochemical, LS003126), 2 mg of l-cysteine (Sigma-Aldrich, C7880) and DNase I (Sigma-Aldrich, D4527) at 34 °C for 40 min. Following tissue digestion, papain was inactivated using a low-ovo solution containing BSA, PBS, Trypsin inhibitor (Worthington Biochemical, LS003086). The brain tissues were dissociated by gently pipetting in low-ovo solution. Following centrifugation, the cell pellet was suspended in PBS and counted. Then, the cell suspension was subjected to magnetic labeling with an Anti-ACSA-2 MicroBead Kit (Miltenyi Biotec, #130–097-679) according to the manufacturer's instructions. Briefly, the cell suspension was blocked with 5–10 µl of FcR blocking reagent per 10^7^ total cells at 4 °C for 10 min; 5–10 µl of Anti-ACSA-2 MicroBeads per 10^7^ total cells was added and incubated at 4 °C for 15 min. The cells were washed by adding 1–2 ml of PBS buffer per 10^7^ total cells and resuspended in 500 µl PBS buffer per 10^7^ total cells. The cell suspension was applied to an MS column (Miltenyi Biotec, #130-042-201) to get purified astrocytes.

### Behavioral tests

Behavioral tests were performed by an individual blinded to experimental groups and measured at 2 months after BCAS surgery.

### Novel object recognition

The Novel Object Recognition test (NOR) was used to evaluate recognition memory. The mice were allowed to adapt to boxes without any object for 15 min in three consecutive days. On day 4, two identical objects were placed in the box, and the mice were allowed to explore freely for 10 min. One hour later, one of the objects was replaced with a new object with different shape and colour. Then mice were allowed another 5 min to explored two objects. Exploratory behavior was recorded. Exploration time was defined as the time spent sniffing and touching objects with the nose or forepaws. The percentage of recognition index was calculated as follows: explore new objects/(explore new objects + explore old objects).

### Y-maze test

Short-term working memory was assessed using a Y-maze device consisting of three identical arms. Before each test, the maze was cleaned with 75% ethanol. Each mouse was allowed to freely explore three arms starting from the center of the maze for 8 min. A camera above the device recorded the sequence in which the mice enter the arm. Spontaneous alternations were defined as entering three consecutive arms in a row (e.g., BAC, BCA, CAB, etc.). The alternation percentage (%) was calculated as followed: (Spontaneous alternations/number of arm entries − 2) × 100. Heatmaps were generated using ANY-maze software (Stoelting, USA).

### Morris water maze test

The Morris Water Maze (MWM) test was used for spatial learning and working memory assessment. The MWM installation consists of a circular tank divided into four quadrants, of which the fourth quadrant has a circular platform 14 cm in diameter. Before the experiment, the tank was filled with water (approximately 1 cm above the platform) which was made opaque with edible titanium dioxide. During the learning phase (days 1–5), mice were allowed to find hidden platforms within 60 s. On the testing phase (day 6), the platform was removed, and the swimming track of mice was recorded. Latency to platform, cross-platform times, time in the target quadrant, swimming speed and heatmaps were automatically generated by ANY-maze software (Stoelting).

### Open field test

The Open Field Test (OFT) was designed to test anxiety-like behavior in mice. The mice were placed in an open box (50 cm × 50 cm × 50 cm) and recorded for 10 min. Time spent in the central and corner regions of mice was measured by ANY-maze software (Stoelting, USA).

### EdU injections and staining

EdU (5-ethynyl-2 '-deoxyuridine) is used to label newly proliferating cells. 14 days before the mice were sacrificed, EdU (5 mg/kg) was intraperitoneally injected once a day for 7 consecutive days. For cells, EdU (10 μM) was added and incubated for 24 h. EdU staining followed the manufacturer’s introduction with Click-iT™ EdU Imaging Kits (Invitrogen).

### PLX5622 feeding procedures

PLX5622 feeding procedure was performed as previously described [19]. For microglia/macrophage depletion, Control Diet (AIN-76A, SYSE Bio, China) and PLX5622 (Based on AIN-76A with 1200 ppm PLX5622, SYSE Bio) was supplied to mice (8 weeks old, 25–30 g body weight) in the diet, starting 14 d prior to surgery and continuing until the end of experiments. One adult mouse consumes about 3.5 g chow diet per day.

### Stereotaxic intracranial injection

Stereotaxic intracranial injection was performed as previously described [20]. The injection site of CC was determined according to the brain map (coordinates: ± 1 mm lateral from the midline; 0.8 mm anterior from bregma; 2.2 mm depth from bregma). After mice were anesthetized by 2.5% avertin, skull was drilled and then 400 nl (200 nl/hemisphere) *Cxcl5*-overexpression AAVs (rAAV-GfaABC1D-*Cxcl5*-mCherry, BrainVTA, China) or control AAVs (rAAV-GfaABC1D-mCherry, BrainVTA, China) were bilaterally injected into the CC. The microelectrode was maintained in situ for 10 min after injection to avoid virus efflux. After the wound was sutured, the mice awakened on thermostatic heat. The final amount of viral injection is 2.4E + 9 vg/ mice.

### Oxygen–glucose deprivation/reoxygenation (OGD/R)

The original medium of primary astrocytes was replaced with glucose-free medium. The cells were then transferred to an anaerobic chamber filled with 5% CO_2_ and 95% N_2_. After 6 h, cells were supplied with normal medium and re-oxygenated for another 12 or 24 h. The cell supernatant was collected for subsequent ELISA detection.

### Assessment of cell viability

Primary microglia and OPCs were cultured in 96-well plates at a density of 1 × 10^4^ cells/well and treated with different doses of rCXCL5 for 24 h. Cell viability was measured by cell counting kit-8 (Dojindo Laboratories, Japan). After 2 h incubation, the optical density (OD) value at 450 nm was detected by microplate reader (Tecan, Switzerland).

### Enzyme linked immunosorbent assay (ELISA)

The concentration of CXCL5 in mouse serum, CC homogenate and cell supernatant was quantified by ELISA kit (CUSABIO, China) according to the manufacturer’s instructions. The OD value at 450 nm was measured using a microplate reader (Tecan, Switzerland) to calculate the level of CXCL5.

### Magnetic resonance imaging (MRI)

MRI images were performed on a 9.4T Bruker MR system (BioSpec 94/20 USR, Bruker) using a 440-mT/m gradient set with an 86-mm volume transit RF coil and a single channel surface head coil. Mice were anesthetized with isoflurane inhalation (2.5–3%) and monitored to maintain constant physiological parameters. Tooth bar and ear bars were used to position mice on a mouse holder for data acquisition. T2-weighted images were acquired using the 2D RARE (rapid acquisition with relaxation enhancement) sequence with the following parameters: repetition time (TR): 2500 ms, echo time (TE): 33 ms, field of view (FOV): 20 mm × 20 mm, matrix: 256 × 256 and 22 adjacent slices of 0.7 mm slice thickness. Diffusion-weighted images were acquired using spin-echo echo-planar imaging (SE-EPI) sequence with the following parameters: Two b-values (b = 0 and 1000 s/mm^2^) along with 30 non-collinear directions, δ = 4.1 ms, Δ = 10.3 ms; TR: 1500 ms, TE: 23.27 ms, FOV: 20 mm × 20 mm, matrix: 128 × 128, and 22 adjacent slices of 0.7 mm slice thickness. Imaging data were converted into NIFTI format using MRIcron. Fractional anisotropy (FA) maps were calculated using the FSL (v.5.0.9) pipeline. FA was calculated after eddy correction and motion correction of each mouse in FSL. The data were analyzed under the guidance of the Institute of High Energy Physics, Chinese Academy of Sciences.

### Compound action potential measurement

The slices (300 μm) of CC was obtained using a vibrating blade microtome (Leica VT1000 s) in oxygenated (95%O_2_/5%CO_2_) iced cutting solution (in mM): 120 Choline Cl^−^, 25 NaHCO_3_, 10 Glucose, 7 MgCl_2_, 5 Na^+^ ascorbate, 3 Na^+^ pyruvate, 2.5 KCl, 1.25 NaH_2_PO_4_, 0.5 CaCl_2_ and incubated in 34 °C cutting solution for 15 min, and then transferred to oxygenated artificial CSF (ACSF) containing (in mM): 124 NaCl, 25 NaHCO_3_, 10 Glucose 2.5 KCl, 2.5 CaCl_2_, 2 MgSO_4_ and 1.25 NaH_2_PO_4_ at room temperature (25 °C) for 1 h before recording. For combined action potential (CAP) measurements, the acute slices containing CC were transferred to the microelectrode array (60MEA200/30iR-ITO), continuously perfused with oxygenated ACSF (2 ml/min) and maintained at 32 °C for recording. The MEA-2100-60-System hardware and LTP-Director software (Multi Channel Systems, Germany) were used for recording CAP in the CC. Input–output curves were generated by varying the intensity of the stimulation from 10 μA to 90 μA (step of 10 μA). LTP-Analyzer (Multi Channel Systems, Germany) software was used for data analysis. Myelinated fiber-response amplitude was defined as the voltage difference from the first peak to the first trough.

### Luxol fast blue (LFB) staining

LFB staining was used to demonstrate myelination [21]. The cryosections were washed with distilled water and stained with 0.1% LFB solution 1 h at 60 °C (Servicebio, China). The slides were then immersed in lithium carbonate solution for 2 s, directly immersed in 70% ethanol for differentiation for 15 s, and washed to complete differentiation. After repeated differentiation, washing and microscopic examination, until the myelin sheath is blue, the background is almost colorless. The sections were then counterstained with eosin. Finally, the sections were dehydrated with sequential anhydrous ethanol baths, cleared through xylene and sealed with neutral resin. The average optical density (AOD) was calculated using ImageJ.

### Statistical analysis

Prism 8 software (GraphPad software, USA) and SPSS software version 22.0 (SPSS Inc., USA) were used for statistical analysis. Data were presented as mean ± SEM. Statistical differences between two groups were determined using Student's t-test or Mann–Whitney test. One-way analysis of variance (ANOVA) followed by Tukey's post-hoc test or Kruskal–Wallis test followed by Tukey's post-hoc test was used to analyse the statistical significance of data with one factor among several groups, and two-way ANOVA followed by Bonferroni's post hoc test was used to analyse the statistical significance of data with two factors. A statistically significant difference was considered at p < 0.05.

## Results

### Chronic cerebral ischemia induces significant up-regulation of CXCL5

**Fig. 1:**
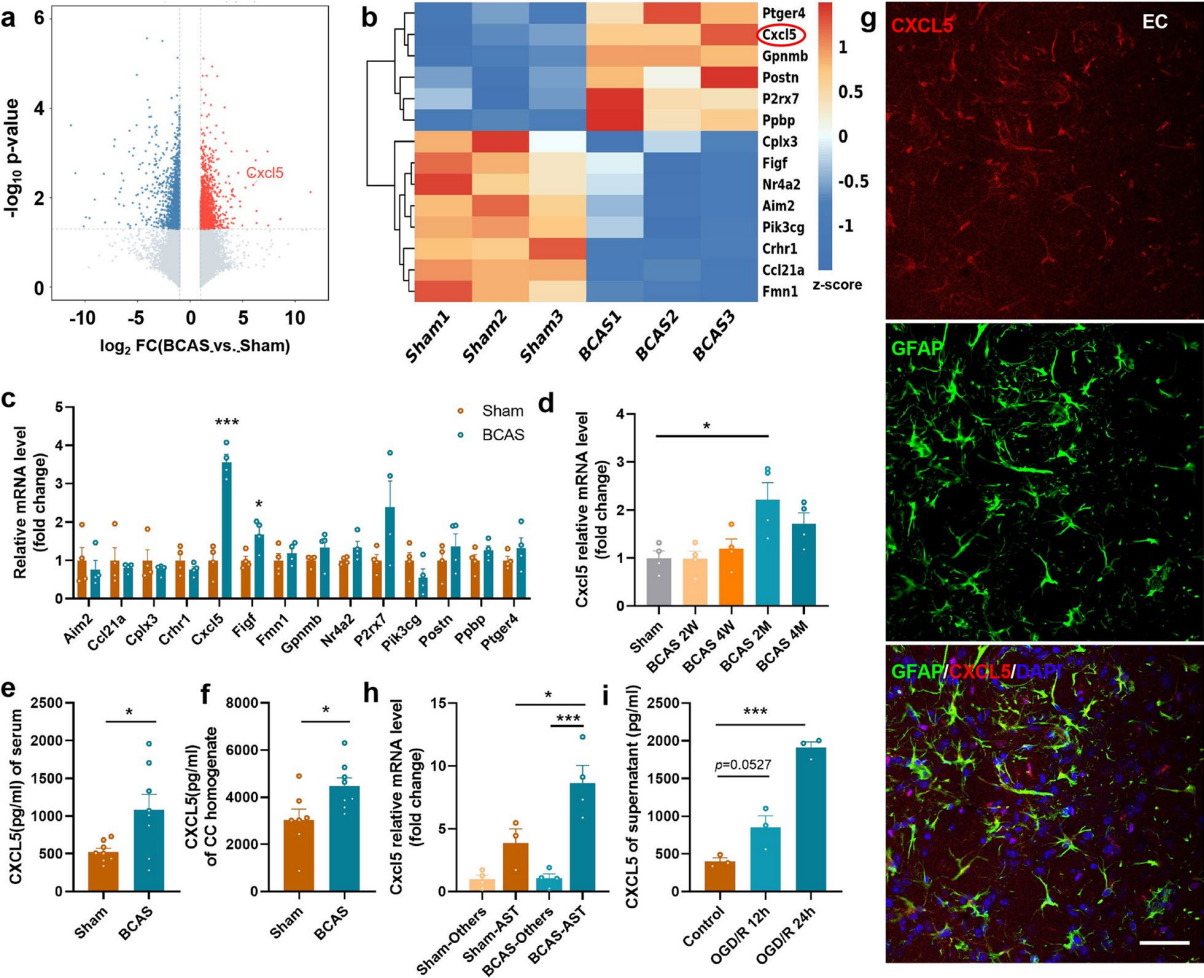
Chronic cerebral ischemia induced significant up-regulation of CXCL5. **a** Volcano plot of the results of CC tissue microarray analysis between the Sham and BCAS groups at 2 months after surgery (fold change > 2; p < 0.05). n = 3 per group. **b** Heatmap of fourteen genes related to immune response form CC tissue microarray analysis. n = 3 per group. **c** The mRNA level of fourteen genes was measured in the Sham and BCAS groups by qPCR. n = 4 per group. **d** The mRNA level of *Cxcl5* in the CC at different time points after BCAS by qPCR. **e** The result of serum CXCL5 concentration in the Sham and BCAS groups by ELISA. n = 8 per group. **f** The result of CXCL5 concentration in the CC homogenate in the Sham and BCAS groups by ELISA. n = 7–8 per group. **g** Representative images of immunostaining against GFAP and CXCL5 in EC in the BCAS groups. (Bar = 50 μm). **h** The mRNA level of *Cxcl5* in the astrocytes (AST) and the other cells (Others) from the CTX and CC in the Sham and BCAS groups by qPCR. n = 4 per group. **i** The result of CXCL5 concentration in the supernatant of primary astrocytes in the control, OGD/R 12 h and OGD/R 24 h groups by ELISA. n = 3 per group. All data were presented as the mean ± SEM. *p < 0.05, ***p < 0.001, ns means no significance. EC: external capsule. 2W, 2 weeks. 2 M, 2 months. Student’s t-test for **c**, **e** and **f**. One-way ANOVA with Tukey’s post-hoc for **d**, **i**, **j**

BCAS mouse model was established to mimic cerebrovascular WMI induced by chronic cerebral hypoperfusion. Gene expression profile was analyzed via transcriptome sequencing analysis using extraction from the CC of mice (Fig. 1a) and subsequent bioinformatics analysis screened out fourteen genes related to immune response (Fig. 1b). Compared with the Sham group, the expression of *Cxcl5* was significantly increased in the CC of BCAS mice (Fig. 1c, p < 0.001). Further, our results demonstrated that the *Cxcl5* mRNA level in the CC was gradually up-regulated and peaked at 2 months after BCAS (Fig. 1d, p < 0.05). Similarly, verified by ELISA, the level of CXCL5 in the BCAS group was remarkably elevated both in the serum (Fig. 1e, p < 0.05) and CC homogenate (Fig. 1f, p < 0.05). Subsequently, we further investigated the source of CXCL5 in the brain after BCAS. Co-staining against CXCL5 and markers for different cells, including neurons (Neuronal nuclei, NeuN), microglia (Ionized calcium binding adaptor molecule 1, Iba-1), primary oligodendrocyte lineage cells (oligodendrocyte transcription factor, Olig2) and astrocytes (glial fibrillary acidic protein, GFAP) indicated that CXCL5 was mainly colocalized with astrocytes (Fig. 1g) while scarcely colocalized with neurons, microglia and oligodendrocyte lineage cells (Additional file 1: Fig. S2). The results suggested that CXCL5 was most likely expressed in astrocytes after BCAS. Then, astrocytes were isolated from the CTX and CC of mice and further confirmed that *Cxcl5* expression in astrocytes was much higher than that in the other cells, especially after BCAS (Fig. 1h, Sham-AST vs. BCAS-AST, p < 0.05; BCAS-Others vs. BCAS-AST, p < 0.001). At last, CXCL5 concentration in the supernatant from primary astrocytes after OGD was significantly higher than that in the control group by ELISA (Fig. 1i, Control vs. OGD/R12h, p = 0.0527; Control vs. OGD/R24h, p < 0.001). Taken together, these results demonstrated that CXCL5 was remarkably elevated in astrocytes in chronic cerebral ischemia.

### Astrocyte-specific *Cxcl5* deficiency alleviates cognitive decline in chronic cerebral ischemia

**Fig. 2:**
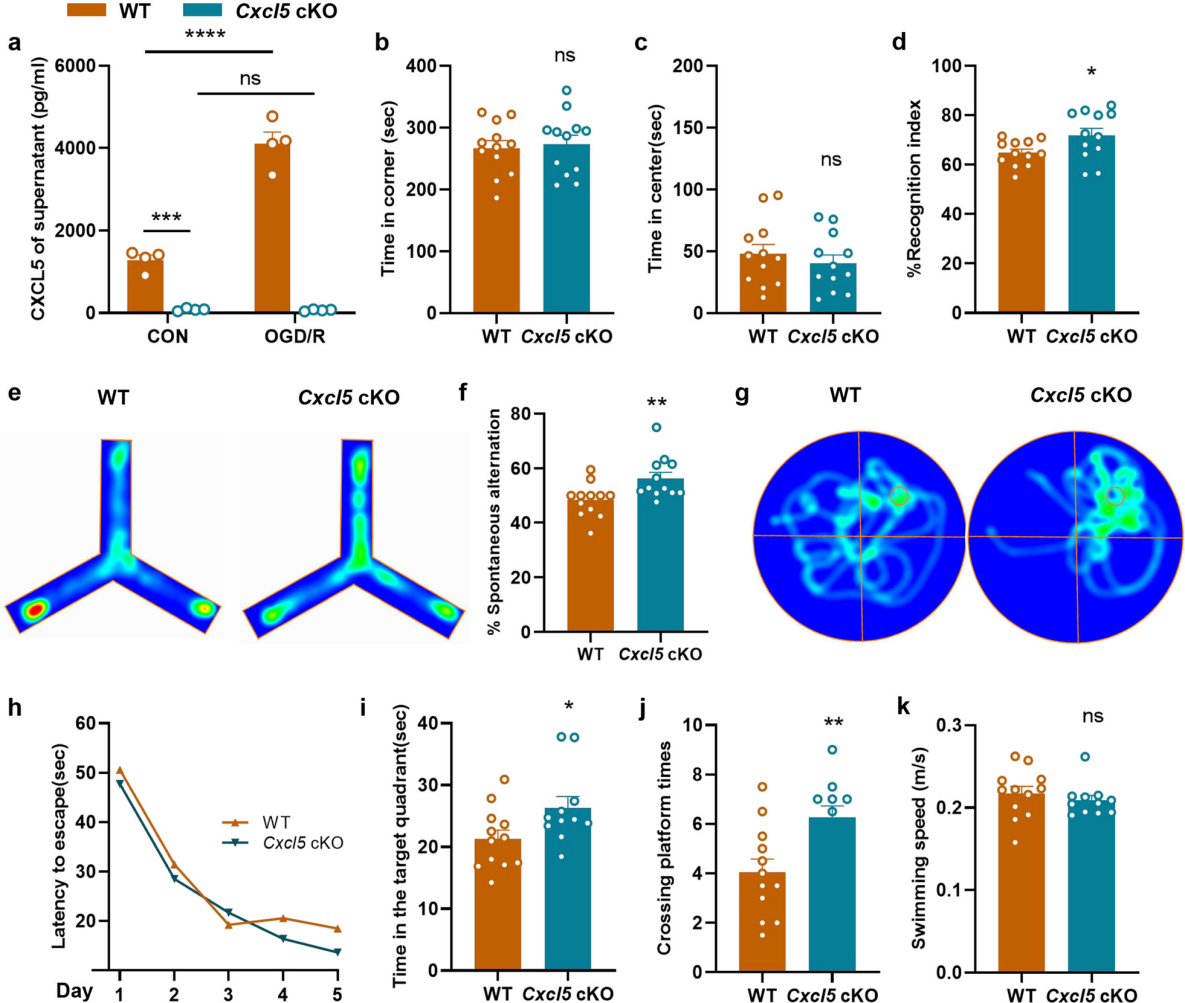
Astrocyte specific *Cxcl5* deficiency alleviated cognitive decline in BCAS model. **a** The result of CXCL5 concentration in the supernatant of primary astrocytes in the WT and *Cxcl5* cKO groups after OGD/R 12 h by ELISA. n = 4 per group. **b**, **c** Results of the OFT showing the time spent in the corner area (**b**) and center area (**c**) in the WT and *Cxcl5* cKO groups at 2 months after surgery. n = 12 per group. **d** Results of the NOR showing the recognition index percentage. n = 12 per group. **e**, **f** Results of Y-maze tests showing representative heatmaps (**e**) and the spontaneous alternation percentage (**f**). n = 12 per group. **g**–**k** Results of the MWM test. Representative heatmaps (**g**), escape latency during the acquisition phase (Days 1–5) (**h**), the time in target quadrant (**i**), the crossing platform times (**j**) and the swimming speed (**k**) of the probe test (Day 6). n = 11–12 per group. All data were presented as the mean ± SEM. *p < 0.05, **p < 0.01, ***p < 0.001, ****p < 0.0001, ns means no significance. Two-way ANOVA with Bonferroni's post hoc test for **a** and **h**. Student’s t-test for **b–d**, **j**. Mann–Whitney test for **f**, **i**, **k**

To further explore the role of astrocytic CXCL5 in WMI, we generated astrocyte conditional *Cxcl5* knockout (cKO) mice. At first, the ELSIA results demonstrated that primary astrocytes from neonatal wild type (WT) mice released much more CXCL5 after OGD treatment, while CXCL5 concentrations in the supernatant of astrocytes from *Cxcl5* cKO mice were nearly undetected at both normal and hypoxic condition (Fig. 2a, CON-WT vs. CON-*Cxcl5* cKO, p < 0.001; CON-WT vs. OGD/R-WT, p < 0.0001), indicating the successful *Cxcl5* deletion in astrocytes in the cKO mice.

No significant basaline differences were found between the same batch of cKO mice and WT littermates at 4 months of age in the behavior tests, including the OFT, NOR, Y-maze and the MWM (Additional file 1: Fig. S3). Two months after BCAS surgery, a series of behavioral tests were performed to determine the impact of astrocytic CXCL5 on the cognitive performance in chronic cerebral ischemia in a same cohort of genotyped mice. No significant differences were found in the corner and center time spent in the OFT between the WT and *Cxcl5* cKO groups (Fig. 2b and c). However, the cKO mice showed improved performance in the NOR test after BCAS (Fig. 2d). Similarly, the Y-maze test showed that the short-term working memory of *Cxcl5* cKO mice were significantly improved compared with WT littermates (Fig. 2e and f). What’s more, as shown by the MWM test, our results demonstrated that astrocytic *Cxcl5* deletion induced improved spatial memory performance after BCAS, as evidenced by the prolonged time spent in the target quadrant (Fig. 2i, p < 0.05) and increased number of platform crossing times (Fig. 2j, p < 0.01), with swimming speed not influenced (Fig. 2k). Nevertheless, the WT and cKO mice displayed comparable latency to find the hidden platform (Fig. 2h) in the MWM test, demonstrating that astrocytic *Cxcl5* deletion might exert no significant impact on learning performance. Taken together, astrocyte specific *Cxcl5* deletion ameliorated cognitive impairment under chronic cerebral hypoperfusion.

### Astrocytic *Cxcl5* deficiency alleviates BCAS-induced white matter injury

**Fig. 3:**
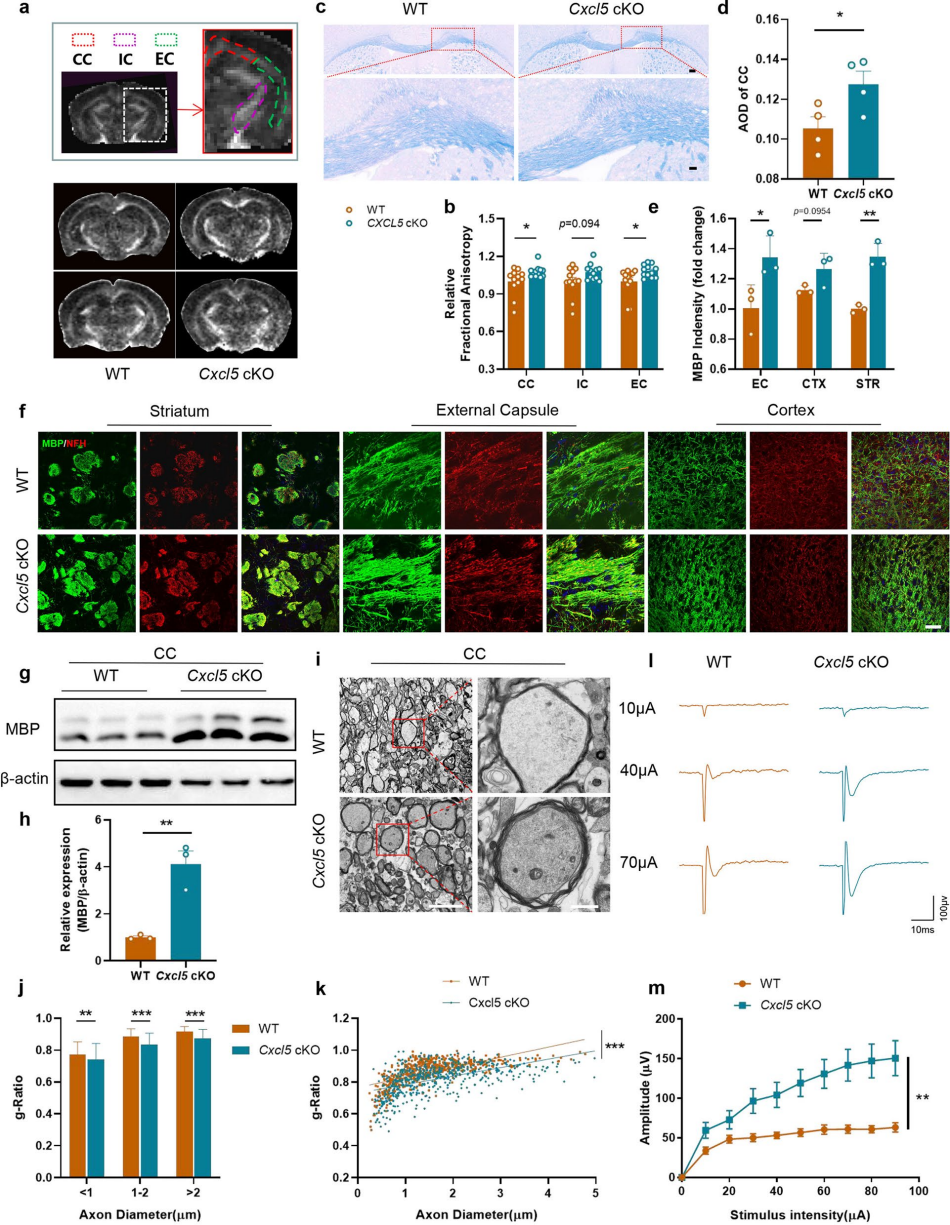
Astrocytic *Cxcl5* deficiency alleviated BCAS-induced white matter damage. **a**, **b** Representative DTI axial views of FA maps (**a**) and quantification of FA values in the CC, IC and EC (**b**) acquired 2 months after BCAS. Two panels above show the areas (CC, IC and EC) of quantification. n = 12 per group. **c**, **d** Representative images of LFB staining (**c**) and quantification of AOD (**d**) in CC. Scale bar = 200 μm (top) and 50 μm (bottom). n = 4 per group. **e**, **f** Representative images for MBP (green) and NFH (red) double immunostaining (**f**) and quantification of the fluorescence intensity of MBP (**e**) in EC, CTX, STR. Scale bar = 50 μm. n = 3 per group. **g**, **h** Representative immunoblot bands of MBP (**g**) and quantification normalized to β-actin (**h**) in the CC. n = 3 per group. **i–k** TEM ultrastructural analyses of myelin integrity in the CC of WT and *Cxcl5* cKO groups at 2 months after BCAS. **i** Representative TEM images in the CC. Scale bar = 5 μm (left) and 10 μm (right). **j** Comparison of the g-ratio of myelinated axons according to axon diameter between WT and *Cxcl5* cKO mice. **k** Scatterplots of the myelin g-ratio as a function of the axon diameter. Axons were selected from 4 mice per group for measurement. n = 545 and n = 543 measured axons for each group. **l**, **m** Representative curves of CAPs of myelinated fibres (**l**) and quantification of the amplitude of evoked CAPs of myelinated fibres (**m**) in the CC. n = 3 mice and 10–12 recordings per group. All data were presented as the mean ± SEM. *p < 0.05, **p < 0.01, ***p < 0.001, ns means no significance. Student’s t-test for **d**, **e**, **h**. Mann–Whitney test for **b**, **j**, **k**. Two-way ANOVA with Bonferroni's post hoc test for **m**

Then, we investigated the influence of astrocytic CXCL5 on WMI at about 2 months after BCAS. Specifically, 9.4T MRI was used to detect the impact of astrocytic CXCL5 on the integrity of white matter, and the key diffusion tensor imaging (DTI) index FA was used to evaluate the microstructure of the white matter. The results indicated that the FA value was higher in *Cxcl5* cKO mice than WT mice after BCAS (Fig. 3a and b, CC, p < 0.05; internal capsule [IC], p = 0.094; the external capsule [EC], p < 0.05), suggesting microstructure in white matter of *Cxcl5* cKO mice was more complete than the controls. Similarly, LFB staining also showed that the white matter damage induced by chronic cerebral ischemia was alleviated in *Cxcl5* cKO mice (Fig. 3c and d). In addition, the improved WMI in *Cxcl5* cKO mice was further confirmed via double staining of the myelin basic protein (MBP) (marker of myelin sheath) and neurofilament-heavy (NFH) (marker of axon) in three white matter-rich brain regions (EC, CTX, and the striatum [STR]) (Fig. 3e and f, EC, p < 0.05; CTX, p = 0.0954; STR, p < 0.01). Immunoblot analysis further illustrated increased the level of MBP protein in the CC of *Cxcl5* cKO than that of WT mice (Fig. 3g and h). In order to directly observe the myelin injury, the CC of mice after BCAS was examined by electron microscopy, and the g-ratio of cKO mice was lower than that of littermate controls (Fig. 3i-k). To test whether structural and functional changes in the white matter lead to axonal conduction delays, we evaluated CAPs of the CC in coronal brain slices from *Cxcl5* cKO mice and control mice after BCAS. The peak of CAPs represented rapid conduction along myelinated axons. The results showed that the peak amplitude was markedly improved in the cKO mice group (Fig. 3l and m, WT + BCAS vs. *Cxcl5* cKO + BCAS, p < 0.01). Taken together, our results revealed that astrocytic CXCL5 aggravated chronic cerebral ischemia-induced WMI.

### CXCL5 exerts no significant direct impact on the fate of OPCs

**Fig. 4:**
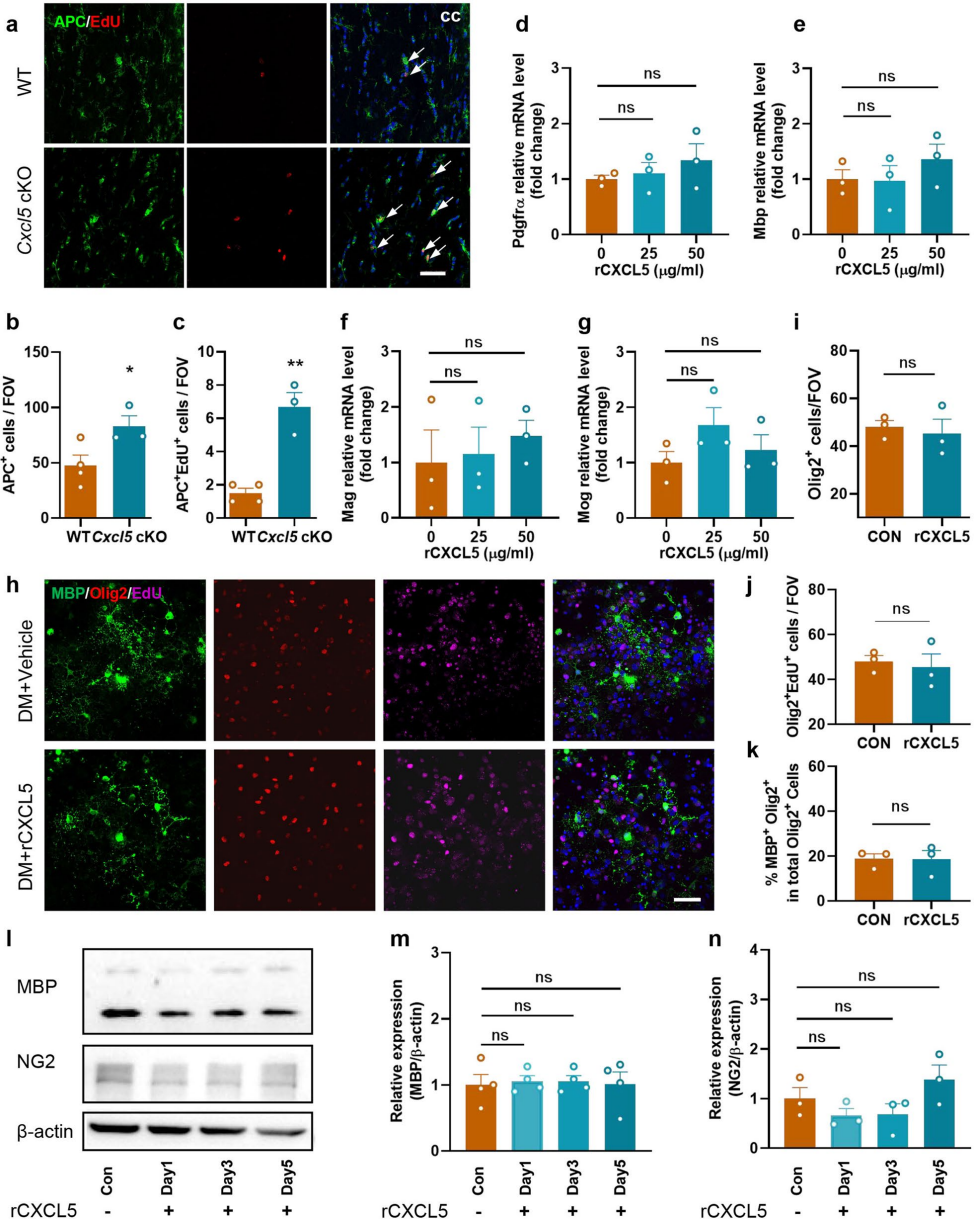
CXCL5 exerted no significant impact on the fate of OPCs. **a**–**c** Representative EdU staining and immunostaining APC (**a**) and quantification of APC^+^ cell (**b**) and EdU^+^APC^+^ cell (**c**) numbers per FOV in the CC in the WT and *Cxcl5* cKO groups at 2 months after surgery. Scale bar = 50 μm. n = 3–4 per group. **d**–**g** The primary OPCs were treated with 25 or 50 μg/ml of rCXCL5 in differentiating medium for 3 days. The mRNA level of OPCs marker Pdgfrα (**d**) and OLs markers Mbp (**e**), Mag (**f**) and Mog (**g**) was measured by qPCR. n = 3 per group. **h**–**k** Primary OPCs were pretreated with rCXCL5 in differentiating medium, and then treated with 10 µM EdU for 24 h. **h** Primary oligodendrocyte lineage cells were labeled by immunofluorescence staining of MBP (green), Olig2 (red) and EdU (pink). Scale bar = 50 μm. **i** Quantification of Olig2^+^ cell numbers in the control and rCXCL5 groups. **j** Quantification of Olig2^+^EdU^+^ cell numbers in the control and rCXCL5 groups. **k** Quantification of the ratio of MBP^+^Olig2^+^ cells among total Olig2^+^ cells in the control and rCXCL5 groups. n = 3 per group. **l**–**n** At the days 1, 3 and 5 during the differentiation phase of OPCs, rCXCL5 was added to the differentiating medium, and finally OLs were collected on day 7. Representative immunoblot bands (**l**) and quantification of MBP (**m**) and NG2 (**n**) normalized to β-actin in OLs. n = 3–4 per group. All data were presented as the mean ± SEM. *p < 0.05, **p < 0.01, ns means no significance. DM: differentiating medium. Student’s t-test for **b**,** c**, **i**, **j** and **k.** One-way ANOVA with Tukey’s post-hoc for **d–g**,** m** and **n**

EdU was intraperitoneally injected 2 months after BCAS to label newly emerging cells [22], with mature oligodendrocytes (OLs) recognized by immunostaining with the anti-APC (adenomatous polyposis coli) antibody, and our results demonstrated an increase in the numbers of APC^+^ OLs and APC^+^EdU^+^ newborn OLs in the CC of *Cxcl5* cKO mice compared with WT mice (Fig. 4a–c). Therefore, we speculated that CXCL5 affects the fate of OPCs.

We further determined whether CXCL5 directly affects the differentiation of OPCs in vitro. rCXCL5 was added to the medium of OPCs, with the optimum concentration (25, 50 μg/ml) determined by the CCK8 test (Additional file 1: Fig. S4a), and our results demonstrated that several characteristic factors marking the differentiation of OPCs showed no meaningful changes after rCXCL5 treatment, including platelet-derived growth factor receptor alpha (Pdgfrα), Mbp, Myelin Associated Glycoprotein (Mag) and Myelin Oligodendrocyte Glycoprotein (Mog) (Fig. 4d–g). In addition, immunofluorescence staining showed that the number of Olig2^+^ cells, EdU^+^Olig2^+^ cells and the ratio of MBP^+^Olig2^+^ cells to total Olig2^+^ cells did not change significantly, suggesting that CXCL5 exerted no direct effect on the proliferation and differentiation of primary OPCs (Fig. 4h–k). To further verify our finding, we added rCXCL5 to primary OPCs at different stages of differentiation, and there were no significant changes in the protein levels of MBP (marker of mature OLs) and neuron-glial antigen 2 (NG2) detected by immunoblot analysis (Fig. 4l–n). Overall, these results told that CXCL5 affects the fate of OL lineage cells indirectly.

### Astrocytic CXCL5 aggravates chronic ischemic WMI in a microglia-dependent manner

**Fig. 5:**
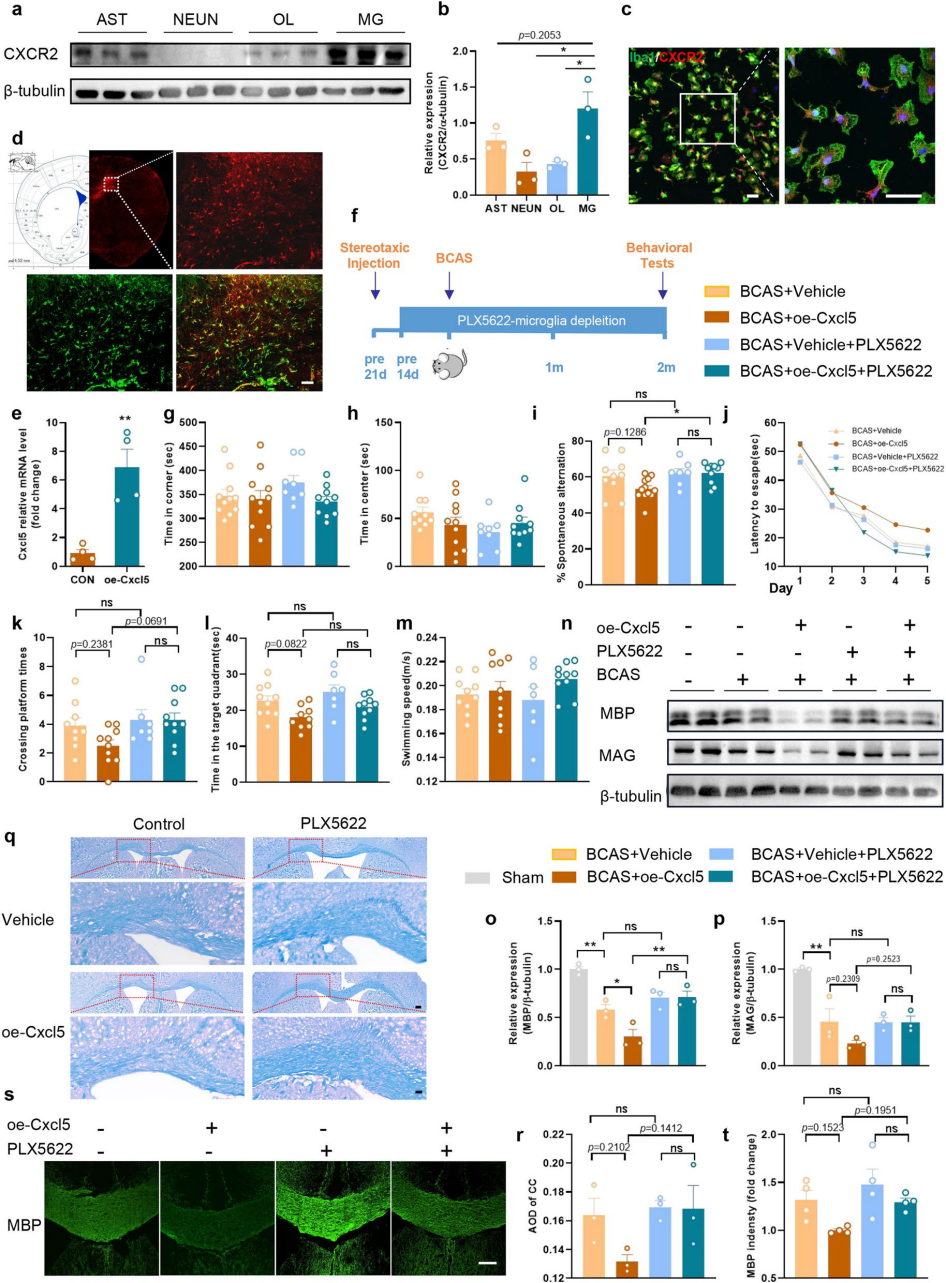
Astrocytic CXCL5 aggravated chronic ischemic WMI in a microglia-dependent manner. **a**, **b** Representative immunoblot bands of CXCR2 and quantification normalized to β-tubulin in primary cells of AST, NEUN, OL and MG. n = 3 per group. **c** Representative images for Iba-1 (green) and CXCR2 (red) double immunostaining in the primary microglia. Scale bar = 50 μm. **d** The rAAV-GFaABC1D-*Cxcl5*-mCherry was injected into the CC. Representative confocal images of mCherry expression within the astrocytes (GFAP). Scale bar = 50 µm (bottom). **e** QPCR analysis of *Cxcl5* expression in the CC at 3 weeks after stereotaxic intracranial injection. n = 4 per group. **f** Experimental design. **g**, **h** Results of the OFT showing the time in the corner and center in four groups. n = 8–11 per group. **i** Results of Y-maze tests showing the spontaneous alternation percentage. n = 7–12 per group. **j**–**m** Results of the MWM test in four groups. **j** Escape latency during the acquisition phase (Days 1–5). Quantification of the crossing platform times (**k**), the time in target quadrant (**l**) and the swimming speed (**m**) of the probe test (Day 6). n = 7–10 per group. **n**–**p** Representative immunoblot bands (**n**) and quantification of MBP (**o**) and MAG (**p**) normalized to β-tubulin in the CC. n = 3 per group. **q**, **r** Representative images of LFB staining in CC and quantification of AOD. Scale bar = 200 μm (top) and 50 μm (bottom). n = 3 per group. **s**, **t** Representative images of immunostaining of MBP and quantification of immunostaining of MBP in the CC. Scale bar = 200 µm. n = 4 per group. All data were presented as the mean ± SEM. *p < 0.05, **p < 0.01, ns means no significance. oe-Cxcl5, overexpression of *Cxcl5*. Two-way ANOVA with Bonferroni's post hoc test for **j**. One-way ANOVA with Tukey’s post-hoc for the others

CXCR2 is the only known functional receptor for CXCL5 currently [23] and we found that CXCR2 was highly expressed in primary microglia (Fig. 5a and b, AST vs. MG, p = 0.2053; NEUN vs. MG, p < 0.05; OL vs. MG, p < 0.05). Consistently, immunofluorescence staining also demonstrated that CXCR2 was highly expressed in microglia (Fig. 5c). Since the important role of microglia in chronic ischemia-induced WMI, we wondered whether astrocytic CXCL5 influences WMI in chronic cerebral ischemia via modulating the activity of microglia.

*Cxcl5* was specifically overexpressed in astrocytes by AAV injection into the CC, and AAV transfection efficiency was evaluated by immunofluorescence staining at 3 weeks after injection (Fig. 5d). Compared with the control group, the mRNA level of *Cxcl5* was significantly increased in the CC of the overexpression group (Fig. 5e). Meanwhile, microglia were removed by feeding PLX5622 (an inhibitor of colony-stimulating factor 1 receptor) to determine whether astrocytic CXCL5 influences white matter damage in a microglia-dependent manner. Dietary intake of PLX5622 for 14 days dramatically reduced the number of microglia in the normal brain (Additional file 1: Fig. S5). Continuation on the PLX5622 diet maintained low numbers of microglia/macrophages in the brain 2 months after BCAS surgery (Fig. 5f).

We randomly divided the same batch of mice into four groups: BCAS + Vehicle, BCAS + Vehicle + PLX5622, BCAS + oe-Cxcl5, BCAS + oe-Cxcl5 + PLX5622, and then we evaluated the cognitive performance of the mice at 2 months after BCAS. Firstly, the OFT illustrated no significant differences in the residence time in the center and corner among all groups (Fig. 5g and h). Then, our results illustrated that decrease in the spontaneous alternations in Y-maze was augmented after astrocytic *Cxcl5* overexpression but mitigated by the depletion of microglia (Fig. 5i). Furthermore, the MWM test illustrated that astrocytic *Cxcl5* overexpression induced mild impairment of spatial memory, while this impairment was rescued when microglia were depleted (Fig. 5j–m). These behavior data suggested that overexpression of *Cxcl5* in astrocytes exacerbate cognitive impairment of BCAS in a microglia-dependent manner. Consistent with the behavior tests, the MBP and MAG protein levels in the CC of mice in each group further confirmed our finding (Fig. 5n-p). Similarly, LFB staining (Fig. 5q and r) and immunofluorescence staining of MBP (Fig. 5s and t) also revealed that astrocytic *Cxcl5* overexpression aggravated white matter damage after BCAS, while depletion of microglia significantly reversed this impact. In summary, our results illustrated that CXCL5 aggravated WMI induced by chronic cerebral ischemia in a microglia-dependent manner.

### CXCL5 hinders microglial clearance of myelin debris

Microglia aggravate WMI mainly by releasing inflammatory cytokines, but they also contribute to the repair process mainly via phagocytosis of myelin debris [24], and we investigated how astrocytic CXCL5 influences WMI via modulating microglial function. The cytotoxicity of rCXCL5 on primary microglia was determined by the CCK8 test, and the optimum concentration of rCXCL5 was ultimately set at 25 μg/ml (Additional file 1: Fig. S4b). Then, we examined the expression of inflammatory cytokines in microglia incubated with rCXCL5 and myelin debris. The results showed that CXCL5 could elevated the level of inos, while no significant changes in il-1β and il-6 were found (Additional file 1: Fig. S6a–c). The expression levels of inflammatory genes were tested in the CC of both the WT and *Cxcl5* cKO mice, and our results indicated that astrocytic CXCL5 exerted no significant impact on the neuroinflammatory response in chronic cerebral ischemia (Additional file 1: Fig. S6d–f).

**Fig. 6:**
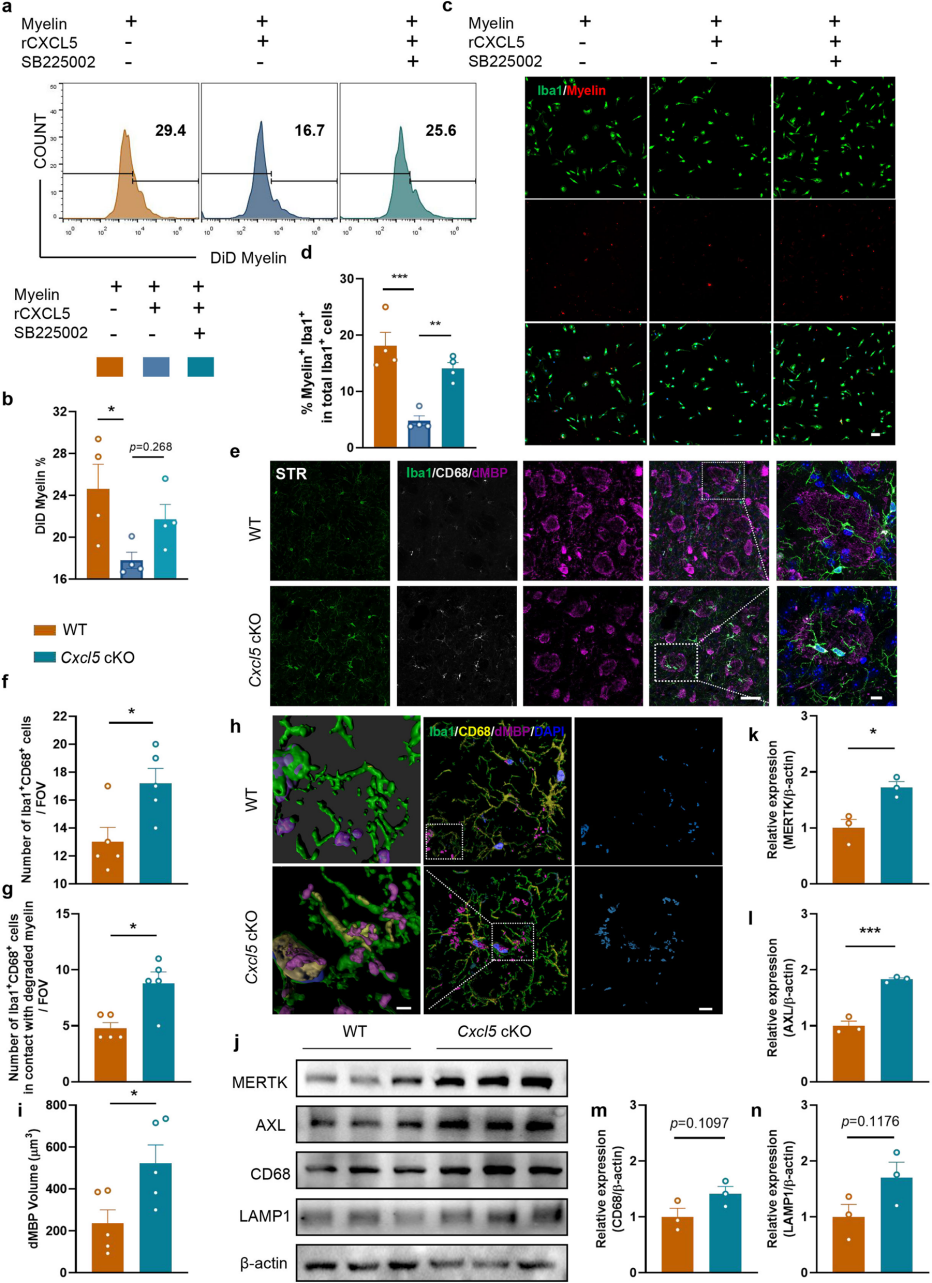
CXCL5 hindered microglia-mediated clearance of myelin debris. **a**, **b** Primary microglia were pretreated with rCXCL5 or rCXCL5 + SB225002 for 0.5 h, then incubated with myelin debris (0.01 mg/ml) stained with DiD for 1 h, primary microglia were collected (**a**) and the ratio of microglia phagocytosing myelin debris (DiD^+^ cells) among total cells were conducted by the flow cytometer (**b**). n = 4 per group. **c**, **d** Primary microglia were pretreated with rCXCL5 or rCXCL5 + SB225002 for 0.5 h, and then incubated with myelin debris (0.01 mg/ml) stained with DiI for 0.5 h. **c** Representative images for Iba-1 (green) and Myelin (red) double immunostaining. Scale bar = 50 μm. **d** Quantification of the ratio of Myelin^+^Iba-1^+^ cells among total Iba-1^+^ cells. n = 4 per group. **e**–**i** Immunostaining of Iba-1 (green)/dMBP (pink)/CD68 (grey)/DAPI (blue) in STR at 2 months after BCAS (**e**). Scale bar = 50 μm (left) and 20 μm (right). Quantification in the FOV in 50 μm images. **f** Quantification of Iba-1^+^CD68^+^ cell numbers in the WT and *Cxcl5* cKO groups. **g** Quantification of Iba-1^+^CD68^+^ cell numbers adhered to dMBP. n = 5 mice per group. **h** Imaris-rendered Iba-1 (green), dMBP (pink) and CD68 (yellow) with the completely localized dMBP-CD68-Iba-1 (engulfed) channel in cyan. Scale bar = 3 μm(left) and 10 μm (middle and right). **i** Quantification of the average volume of dMBP in per Iba-1^+^CD68^+^ cell in the WT and *Cxcl5* cKO groups. n = 5 per group. **j–n** Representative immunoblot bands (**j**) and quantification of MERTK (**k**), AXL (**l**), CD68 (**m**) and LAMP1 (**n**) normalized to β-actin in the CC. n = 3 per group. All data were presented as the mean ± SEM. *p < 0.05, **p < 0.01, ***p < 0.001, ns means no significance. One-way ANOVA with Tukey’s post-hoc for **b** and** d**. Mann–Whitney test for **g**. Student’s t-test for the others

Subsequently, flow cytometry was used to detect the effect of CXCL5 on the phagocytosis of microglia. We found that rCXCL5 treatment reduced the ability of microglia to phagocytose myelin debris, whereas SB225002 (a CXCR2 antagonist) reversed this effect (Fig. 6a and b), which was further confirmed by the immunofluorescence staining (Fig. 6c and d). In addition, astrocytes extracted from *Cxcl5* cKO mice or their WT littermates were co-cultured with primary microglia and then myelin debris was added. Our results showed an enhanced phagocytic capacity in primary microglia when co-cultured with astrocytes from the cKO mice (Additional file 1: Fig. S7a–c). To determine the impact of CXCL5 on microglial phagocytosis of myelin debris in vivo, triple-labeling of Iba-1, CD68 (Cluster of Differentiation 68, a well-described marker of microglial phagolysosomes) and dMBP (damaged myelin basic protein) was performed. Our results demonstrated that the number of Iba-1^+^CD68^+^ microglia in the STR was increased in *Cxcl5* cKO group compared with WT group (Fig. 6e and f). Similarly, cKO group displayed enhanced spatial contact between CD68-positive microglia and dMBP (Fig. 6g). By 3D-confocal, we confirmed that the microglia could phagocytose myelin debris (Fig. 6h). Moreover, approximately twice the volume of dMBP was engulfed within CD68 in cKO microglia compared with WT microglia on average (Fig. 6i). In addition, the levels of phagocytosis related proteins (AXL receptor tyrosine kinase [AXL], Mer receptor tyrosine kinase [MERTK], lysosomal-associated membrane protein 1 [LAMP1] and CD68) were increased in the CC of *Cxcl5* cKO mice after BCAS (Fig. 6j–n). In summary, these results demonstrated that astrocytic CXCL5 hindered microglial phagocytosis of myelin debris.

## Discussion

WMI is an invariant feature of CSVD, which induces CSVD-related cognitive decline [25, 26]. Glial cells, including astrocytes and microglia, have been widely reported to participate in CSVD-induced WMI, both in the damage and repair process [13, 27, 28]. Thus, key molecules modulating the function of the glial cells might be novel therapeutic targets for CSVD. Here, we found that CXCL5 was up-regulated mainly in astrocytes in the CC region in a mouse model of BCAS, a widely accepted animal model of CSVD with WMI and cognitive decline. Interestingly, astrocyte-specific deletion of *Cxcl5* alleviated WMI and cognitive decline in BCAS mice. Moreover, astrocyte-derived CXCL5 exerted its function of aggravating WMI and cognitive decline in BCAS mice in a microglia-dependent manner. In addition, CXCL5 inhibited the function of microglia in clearing myelin debris.

CXCL5 is an inducible cytokine in glial cells following proinflammatory stimulation. CXCL5 exerts its biological function via interaction with the CXCR2 receptor. It has been widely reported that CXCL5 promotes the chemotaxis of neutrophils [29–31]. CXCL5 has been reported to influence the glial cells-mediated neuroinflammation and the WMI in CNS diseases. However, the role of CXCL5 in CSVD remains uninvestigated. Here, using transcriptome sequencing analysis, we found that CXCL5 was significantly elevated in the CC area of BCAS mice, and we further revealed an up-regulated level of CXCL5 in the serum of BCAS mice. In addition, we found that CXCL5 was mainly released by astrocytes in the BCAS model. Functionally, our study revealed that astrocyte specific *Cxcl5* knockout improved chronic cerebral ischemia-induced WMI and related cognitive decline, which was partly attributed to the enhanced clearance of myelin debris mediated by microglia. In vitro studies confirmed that rCXCL5 protein significantly inhibited microglial phagocytosis of myelin debris. CXCL5 has been reported to induce astrocytosis and aggravate BBB injury [10], thus the influence of CXCL5 on astrocytes and endothelial cells in CSVD needs further investigation.

The debris derived from damaged myelin and brain cells inhibit the differentiation of OPCs [32, 33], which is a major obstacle of the remyelination process in CSVD. The clearance of these debris mediated by mononuclear phagocytosis, especially microglial phagocytosis, improves the microenvironment for OPCs differentiation and contributes to the restoration of WMI [34, 35]. Interestingly, microglia do not play their function of phagocytosis independently, but are influenced by other brain cells, especially astrocytes. Astrocytes and microglia manifest close crosstalk in the normal brain and in the progression of CNS diseases, and astrocytes have been reported to modulate microglial phagocytosis in various CNS diseases. For example, He et al. defined an astrocyte-microglia circuit mediated by the IL-33-ST2-AKT signaling axis supporting microglial metabolic adaptation and phagocytic function, the disruption of which impairs neurodevelopment [36]. Vainchtein et al. also reported that astrocyte-derived IL-33 promoted microglial engulfment of synaptic proteins to restrict excitatory synapse numbers during development [37]. Currently, the crosstalk between astrocytes and microglia in CSVD remains poorly studied. In this study, we revealed that CXCL5-CXCR2 signaling mediated a novel astrocyte-microglia circuit in CSVD, as astrocyte-derived CXCL5 aggravated WMI and cognitive decline via inhibiting microglial phagocytosis of myelin debris after binding to microglial CXCR2. Moreover, deletion of microglia was found to rescue the exacerbated WMI and cognitive decline induced by astrocytic *Cxcl5* overexpression.

Microglial phagocytosis of myelin debris is a sophisticated process, involving multiple key molecules. Firstly, once myelin is damaged, “eat me” signal, like phospholipid phosphatidylserine, is exposed. After sensing by microglia, soluble opsonins, like galectin-3 (Gal-3), growth arrest-specific protein 6 (Gas6) and milk fat globule EGF factor 8 (MFG-E8), are released and bind to the “eat me” signal. At last, the opsonins are recognized and bond by the phagocytic receptors on microglia, like AXL and MERTK [34, 35, 38]. In this study, CXCL5 was proven to inhibit microglial phagocytosis of myelin debris in BCAS mice, which might be attributed to the reduction of phagocytosis-associated molecules, such as AXL and MERTK, while the potential mechanism underlying this action of CXCL5 still need further investigation. In addition, BCAS was used as an animal model of CSVD mimicking chronic ischemic hypoperfusion. Although WMI and related cognitive decline were successfully induced, there exists obvious defects in this model. Most importantly, the cerebral hypoperfusion is artificially caused by the stenosis of large arteries instead of small vessels in BCAS, since which is induced by narrowing of the carotid artery with microcoils [39]. Moreover, the BCAS model lacks the major factors associated with CSVD, aging and hypertension [40, 41]. Thus, more suitable models are needed to explore the roles of CXCL5 in CSVD in the future. More importantly, further studies are needed to investigate the clinical impacts of CXCL5 on CSVD, like determining whether the level of CXCL5 in the blood of CSVD patients is elevated and the correlation between the level of CXCL5 and the prognosis of CSVD patients.

## Conclusions

Our study revealed that astrocyte-derived CXCL5 aggravated WMI and cognitive decline by inhibiting microglial phagocytosis of myelin debris, suggesting a novel astrocyte-microglia circuit mediated by CXCL5-CXCR2 signaling in chronic cerebral ischemia.

## Supplementary Information

**Additional file 1: Figure S1.** Construction strategy diagram of specific *Cxcl5* knockout in astrocyte of GFAP-Cre: *Cxcl5*^fl/fl^ mice. **Figure S2.** CXCL5 was co-stained with neurons, oligodendrocyte lineage cells or microglia in the BCAS group. **Figure S3.** *Cxcl5* cKO and WT littermates showed no significant baseline neurobehavioural differences. **Figure S4.** Effects of rCXCL5 on cell viability. **Figure S5.** Dietary intake of PLX5622 dramatically reduced the number of microglia in the brain. **Figure S6.** Effects of CXCL5 on microglial inflammatory cytokines in vitro and in vivo. **Figure S7.** Phagocytosis of microglia in co-culture with astrocytes. **Table S1.** Primer sequences for identification of *Cxcl5* cKO mice. **Table S2.** Details of the compounds. **Table S3.** Details of the primer sequences.

## Data Availability

The microarray data are deposited in the Gene Expression Omnibus (GEO) database with the accession number GEO: GSE220106. All other data supporting the findings of this study are available from the corresponding author upon reasonable request.

## Abbreviations

AAV: Adeno-associated virus
AOD: Average optical density
AXL: AXL receptor tyrosine kinase
BBB: Blood brain and barrier
BCAS: Bilateral carotid artery stenosis
CAPs: Compound action potentials
CC: Corpus callosum
CD68: Cluster of Differentiation 68
cKO: Conditional knockout
CNS: Central nervous system
CSVD: Cerebral small vascular disease
CTX: Cortex
CXCL5: C-X-C motif chemokine ligand 5
CXCR2: C-X-C motif chemokine receptor 2
DM: Differentiating medium
dMBP: Damaged myelin basic protein
DTI: Diffusion Tensor Imaging
EC: External capsule
FA: Fractional anisotropy
FOV: Field of view
IC: Internal capsule
LAMP1: Lysosomal-associated membrane protein 1
LFB: Luxol Fast Blue
Mag: Myelin Associated Glycoprotein
MBP: Myelin basic protein
MERTK: Mer receptor tyrosine kinase
Mog: Myelin Oligodendrocyte Glycoprotein
MRI: Magnetic resonance imaging
MWM: Morris Water Maze
NFH: Neurofilament-heavy
NG2: Neuron-glial antigen 2
NLRP3: NOD-like receptor thermal protein domain associated protein 3
OD: Optical density
OGD/R: Oxygen–glucose deprivation/reoxygenation
OLs: Oligodendrocytes
OPCs: Oligodendrocyte progenitor cells
OPC/OLs: Primary oligodendrocyte lineage cells
Pdgfrα: Platelet-derived growth factor receptor alpha
PMG: Primary microglia
qPCR: Quantitative real-time PCR
rCXCL5: Recombinant CXCL5
STR: Striatum
WMI: White matter injury
WT: Wild type
