# Supplementary material for: Astrocytic CXCL5 hinders microglial phagocytosis of myelin debris and aggravates white matter injury in chronic cerebral ischemia
Source: J Neuroinflammation. 2023 May 3;20:105. doi: 10.1186/s12974-023-02780-3 (PMC10155379; doi:10.1186/s12974-023-02780-3)
Supplement: Supplementary file 1 — Additional file 1: Figure S1. Construction strategy diagram of specific Cxcl5 knockout in astrocyte of GFAP-Cre: Cxcl5fl/fl mice. Figure S2. CXCL5 was co-stained with neurons, oligodendrocyte lineage cells or microglia in the BCAS group. Figure S3. Cxcl5 cKO and WT littermates showed no significant baseline neurobehavioural differences. Figure S4. Effects of rCXCL5 on cell viability. Figure S5. Dietary intake of PLX5622 dramatically reduced the number of microglia in the brain. Figure S6. Effects of CXCL5 on microglial inflammatory cytokines in vitro and in vivo. Figure S7. Phagocytosis of microglia in co-culture with astrocytes. Table S1. Primer sequences for identification of Cxcl5 cKO mice. Table S2. Details of the compounds. Table S3. Details of the primer sequences. [file 12974_2023_2780_MOESM1_ESM.docx]

**Supplemental material for**

**Astrocytic CXCL5 hinders microglial phagocytosis of myelin debris and aggravates white matter injury in chronic cerebral ischemia**

Qian Cao, Jian Chen, Zhi Zhang, Shu Shu, Yi Qian, Lixuan Yang, Lushan Xu, Yuxin Zhang, Xinyu Bao, Shengnan Xia, Haiyan Yang, Yun Xu, Shuwei Qiu

**This file includes:**

**Figure S1 to S5**

Figure S1. Construction strategy diagram of specific *Cxcl5* knockout in astrocyte of GFAP-Cre: *Cxcl5*^fl/fl^ mice**.**

Figure S2. CXCL5 was co-stained with neurons, oligodendrocyte lineage cells or microglia in the BCAS group.

Figure S3. *Cxcl5* cKO and WT littermates showed no significant baseline neurobehavioural differences.

Figure S4. Effects of rCXCL5 on cell viability.

Figure S5. Dietary intake of PLX5622 dramatically reduced the number of microglia in the brain.

Figure S6. Effects of CXCL5 on microglial inflammatory cytokines *in vitro* and *in vivo*.

Figure S7. Phagocytosis of microglia in co-culture with astrocytes.

**Table S1 to S3**

Table S1. Primer sequences for identification of *Cxcl5* cKO mice.

Table S2. Details of the compounds.

Table S3. Details of the primer sequences.

**Additional Figures:**

**
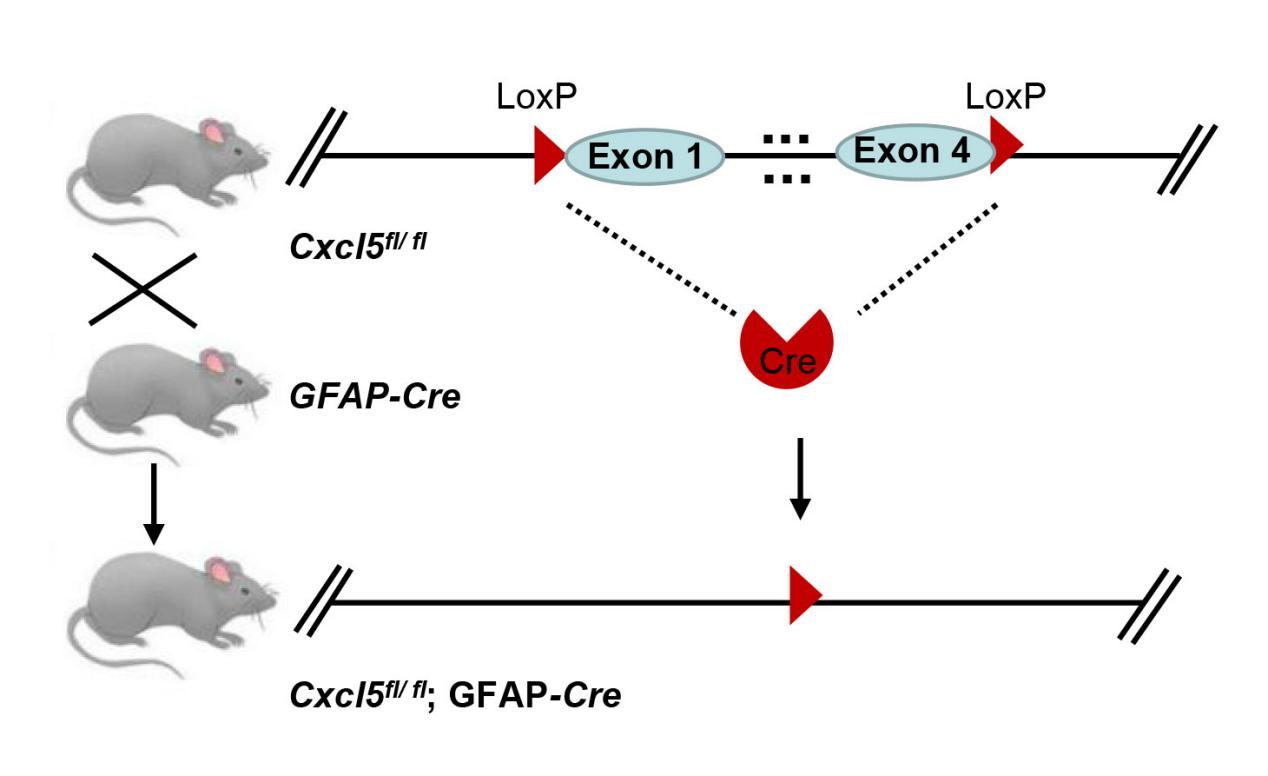
**

**Additional Figure S1. Construction strategy diagram of specific *Cxcl5* knockout in astrocyte of GFAP-Cre: *Cxcl5*^fl/fl^ mice.**


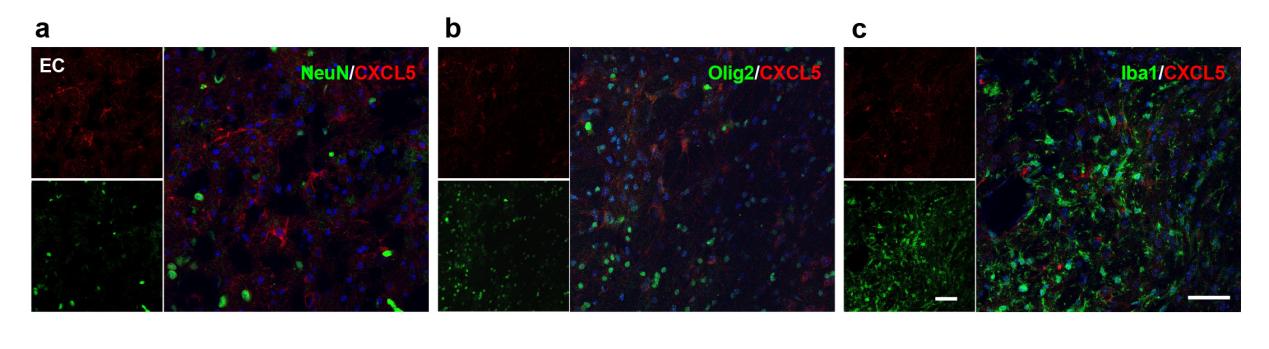


**Additional Figure S2. CXCL5 was co-stained with neurons, oligodendrocyte lineage cells or microglia in the BCAS group.**

Representative images of immunostaining against NeuN (a), Olig2 (b), Iba-1 (c) and CXCL5 in the BCAS groups. (Bar = 50 μm). EC, external capsule.


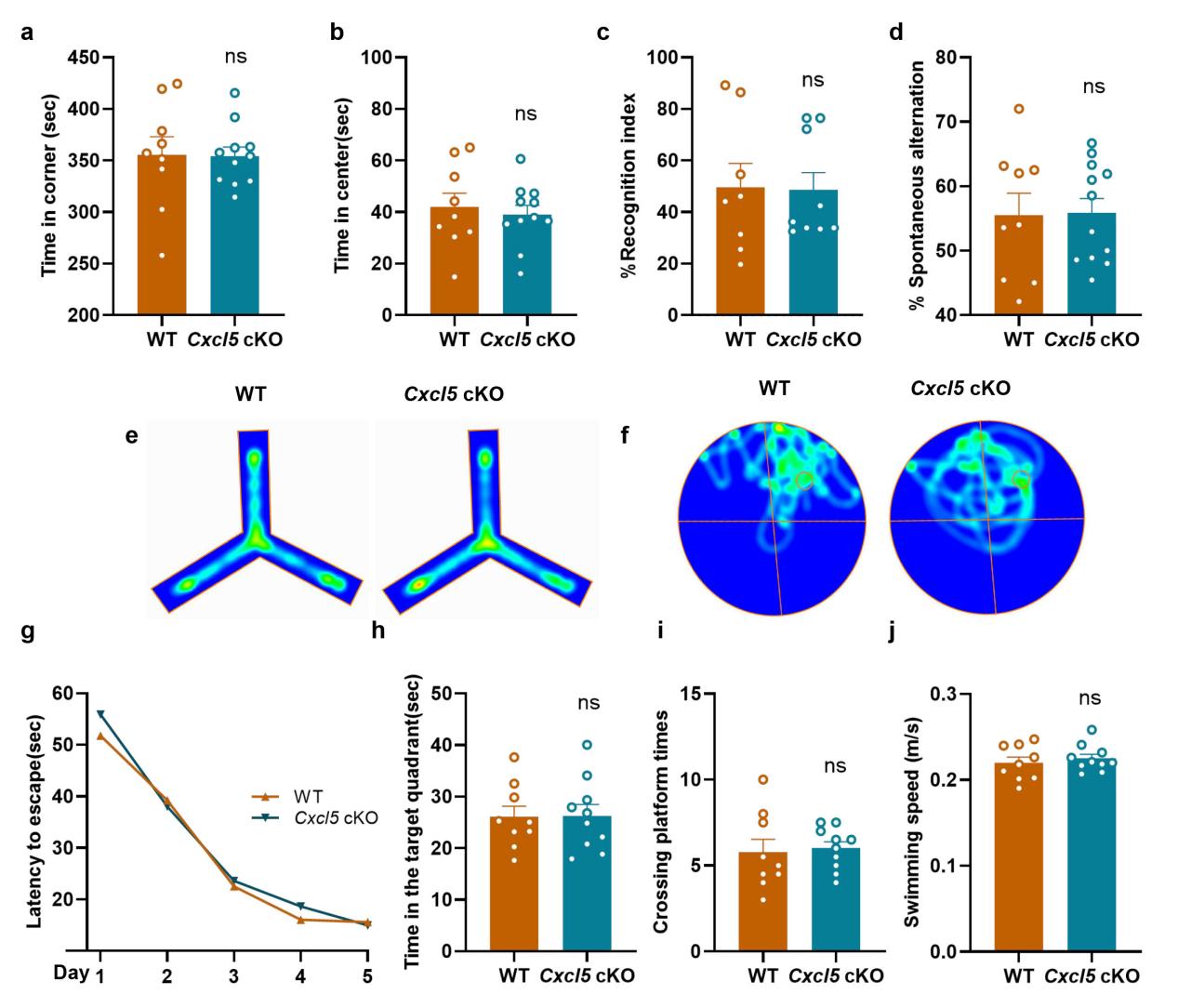


**Additional Figure S3. *Cxcl5* cKO mice and WT littermates were no significant basal differences in the neurobehavioral tests.**

**(a-b)** Results of the OFT showing the time spent in the corner area (a) and center area (b) in the WT and Cxcl5 cKO groups. n = 9-11 per group.

**(c)** Results of the NOR showing the recognition index percentage. n = 8-9 per group.

**(d-e)** Results of Y-maze tests showing the spontaneous alternation percentage (d) and representative heatmaps (e). n = 9-12 per group.

**(f-j)** Results of the MWM test. Representative heatmaps (f), escape latency during the acquisition phase (Days 1–5) (g), the time in target quadrant (h), the crossing platform times (i) and the swimming speed (j) of the probe test (Day 6). n = 9-12 per group.

All data were presented as the mean ± SEM. ns means no significance. Two-way ANOVA with Bonferroni's post hoc test for **g**. Mann–Whitney test for **c**.Student’s t-test for the others.


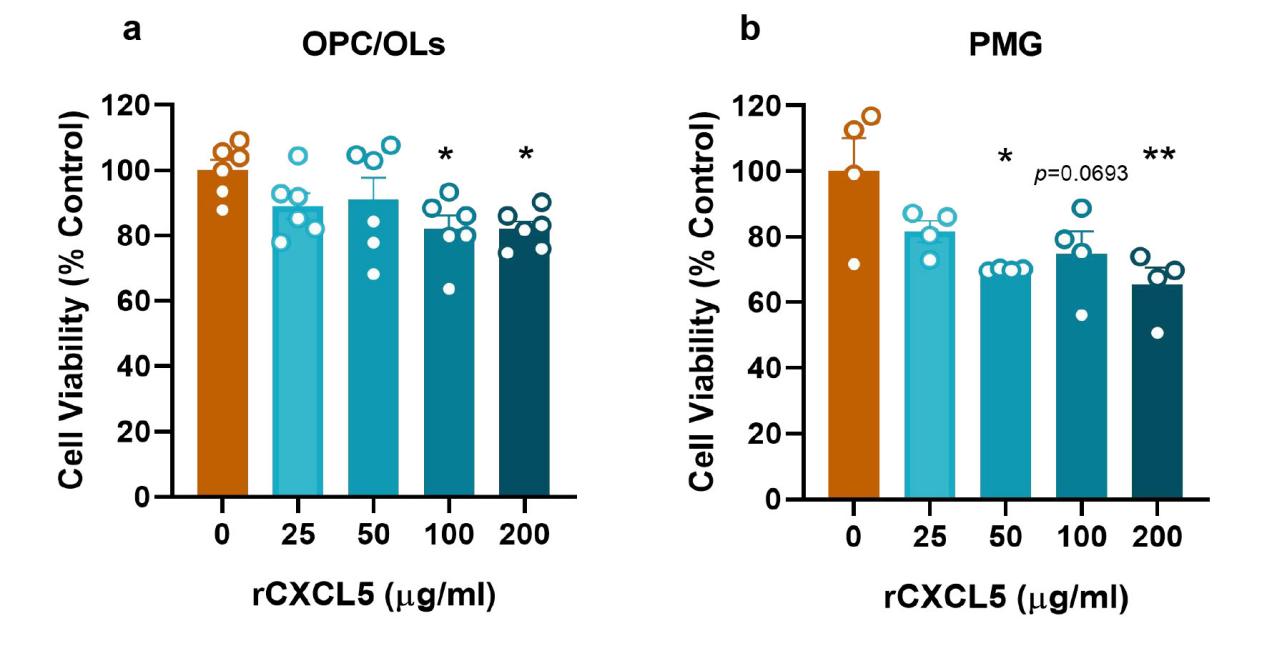


**Additional Figure S4. Effects of rCXCL5 on cell viability.**

**(a)** Primary oligodendrocyte lineage cells were treated with different doses of rCXCL5 (25, 50, 100 or 200 μg/ml). 24 h later, the CCK-8 assay was added to evaluate cell viability. n = 6 per group.

**(b)**Primary microglia were treated with different doses of rCXCL5 (25, 50, 100 or 200 μg/ml). 24 h later, the CCK-8 assay was added to evaluate cell viability. n = 4 per group.

All data were presented as the mean ± SEM. *p < 0.05, ns means no significance. PMG, primary microglia; OPC/OLs, Primary oligodendrocyte lineage cells. One-way ANOVA with Tukey’s post-hoc.


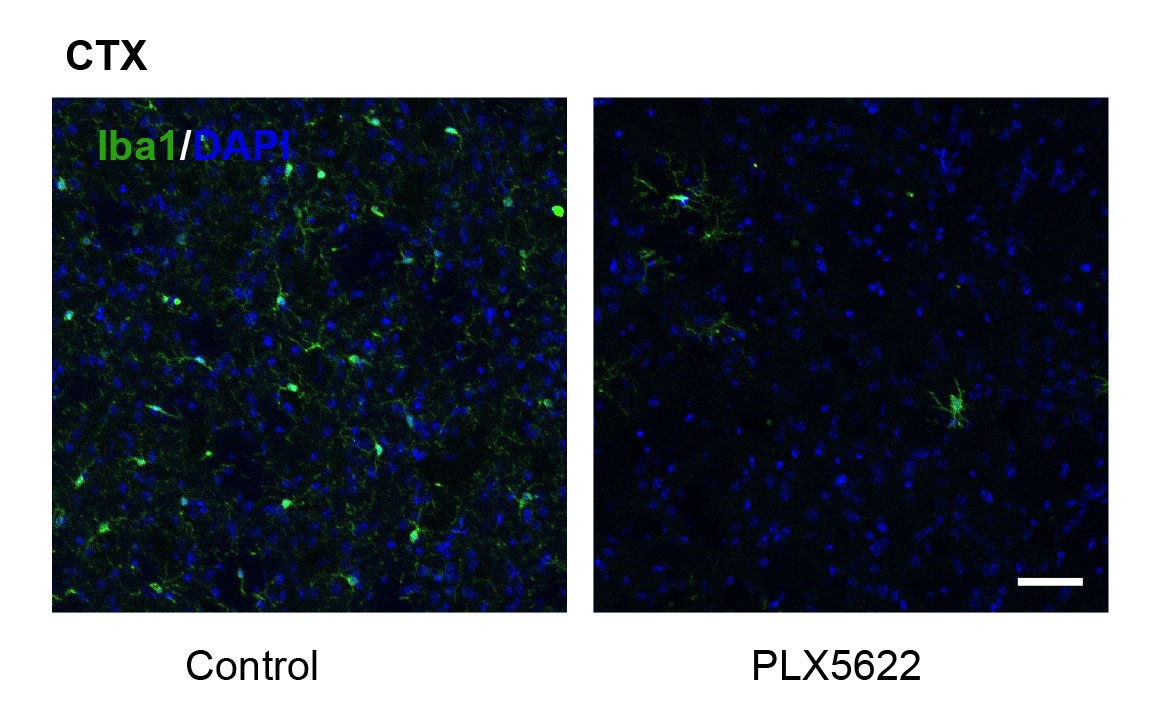


**Additional Figure S5. Dietary intake of PLX5622 dramatically reduced the number of microglia in the brain.**

Representative images of immunostaining of Iba-1 in control and PLX5622 groups at 2 months after surgery. Scale bar = 50 µm. CTX, cortex.


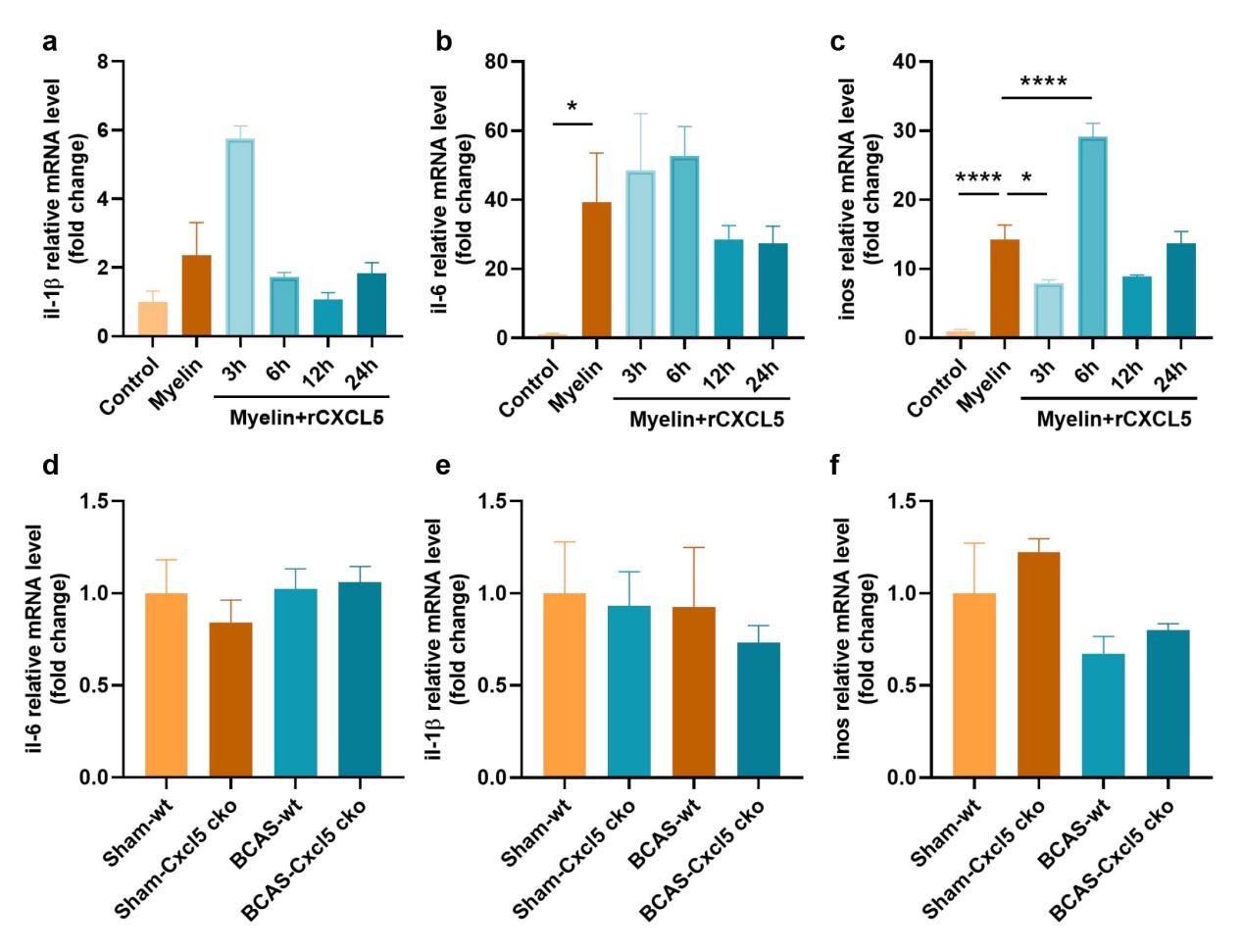


**Additional Figure S6. Effects of CXCL5 on microglial inflammatory cytokines *in vitro* and *in vivo*.**

**(a-c)** Primary microglia were pretreated with rCXCL5 for 0.5 h, then incubated with myelin debris for different time points. The expression of il-1β (a), il-6 (b) and inos (c) were measured at different time points by qPCR. n = 3-4 per group.

**(d-f)** The mRNA level of il-1β (d), il-6 (e) and inos (f) were measured in CC in the WT and *Cxcl5* cKO groups by qPCR. n = 4-5 per group.

All data were presented as the mean ± SEM. *p < 0.05, ***p < 0.001, ****p < 0.0001, ns means no significance. Kruskal–Wallis test with Tukey’s post-hoc for **a**. One-way ANOVA with Tukey’s post-hoc for the others.


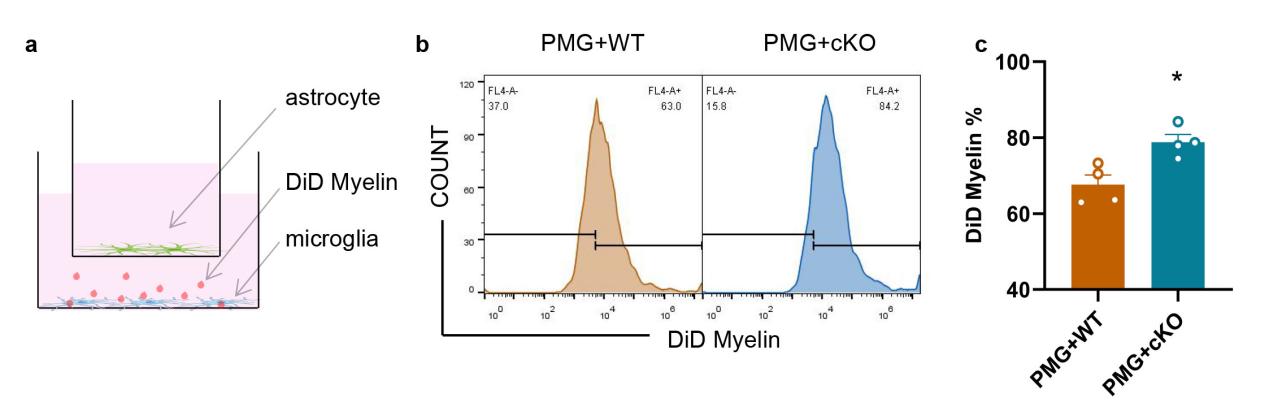
**Additional Figure S7. Phagocytosis of microglia in co-culture with astrocytes.**

(a) Experimental design. (b-c) Primary microglia (PMG) were co-cultured with primary astrocytes from astrocyte conditional *Cxcl5* knockout (cKO) mice and their WT controls for 24 h, then incubated with myelin debris (0.05 mg/ml) stained with DiD for 1 h, primary microglia were collected (b) and the ratio of microglia phagocytosing myelin debris (DiD^+^ cells) among total cells were conducted by the flow cytometer (c). n = 4 per group. All data were presented as the mean ± SEM. *p < 0.05. Student’s t-test.

**Supplementary Tables：**

**Supplementary Table 1.**

Primer sequences for identification of *Cxcl5* cKO mice

| Primer Name | Sequence |
| --- | --- |
| JS09603-Cxcl5-3wt-tF1 | GGCAAATTCACAGTTTGGCAC |
| JS09603-Cxcl5-3wt-tR1 | TAAGCCAGGTTGCTGACTTTAGG |
| H11-tF2 | ATGCCCACCAAAGTCATCAGTGTAG |
| 3722-mGFAP-iCre-KI-5tR1 | TCAGCAACGCTGGAGAATCCC |
| H11-wt-tF1 | CAGCAAAACCTGGCTGTGGATC |
| H11-wt-tR1 | ATGAGCCACCATGTGGGTGTC |

**Supplementary Table 2.**

Details of the compounds

| **Compound** | **Manufacturer** | **Catalog number** |
| --- | --- | --- |
| Rabbit anti-NG2 | Millipore | AB5320 |
| Rabbit anti-dMBP | Millipore | AB5864 |
| Rabbit anti-Olig2 | Millipore | ABN899 |
| Mouse anti-APC | Millipore | OP80 |
| Rabbit anti-MBP | Abcam | ab7349 |
| Mouse anti-MAG | Abcam | ab89780 |
| Rabbit anti-MERTK | Abcam | ab95925 |
| Goat anti-Iba1 | Abcam | ab178846 |
| Mouse anti-NeuN | Abcam | ab104224 |
| Rabbit anti-NFH | Abcam | ab8135 |
| Rat anti-CD68 | Abcam | ab53444 |
| Rabbit anti-LAMP1 | Abcam | ab24170 |
| Rabbit anti-β-Actin | Bioworld | AP0060 |
| Rabbit anti-β-Tubulin | Bioworld | AP0064 |
| Goat anti-Axl | R&D Systems | AF854 |
| Rabbit anti-CXCL5 | Biorbyt | orb389659 |
| Mouse anti-GFAP | Cell Signaling Technology | 3670S |
| Rabbit anti-CXCR2 | Thermo Fisher Scientific | PA5-100951 |
| Recombinant Mouse LIX Protein | R&D Systems | 433-MC |

**Supplementary Table 3.**

Details of the primer sequences

| Primer Name | Sequence |
| --- | --- |
| Cxcl5 forward | TCCAGCTCGCCATTCATGC |
| Cxcl5 reverse | TTGCGGCTATGACTGAGGAAG |
| Gapdh forward | AGGTCGGTGTGAACGGATTTG |
| Gapdh reverse | TGTAGACCATGTAGTTGAGGTCA |
| Mog forward | AGCTGCTTCCTCTCCCTTCTC |
| Mog reverse | ACTAAAGCCCGGATGGGATAC |
| Mag forward | CTGCCGCTGTTTTGGATAATGA |
| Mag reverse | CATCGGGGAAGTCGAAACGG |
| Mbp forward | GACCATCCAAGAAGACCCCAC |
| Mbp reverse | GCCATAATGGGTAGTTCTCGTGT |
| Pdgfra Forward | TCCATGCTAGACTCAGAAGTCA |
| Pdgfra Reverse | TCCCGGTGGACACAATTTTTC |
| Aim2 Forward | GTCACCAGTTCCTCAGTTGTG |
| Aim2 Reverse | CACCTCCATTGTCCCTGTTTTAT |
| Ccl21a Forward | GTGATGGAGGGGGTCAGGA |
| Ccl21a Reverse | GGGATGGGACAGCCTAAACT |
| Fmn1 Forward | CAGCAGCCAAACGAACATCC |
| Fmn1 Reverse | CTCCTGCAACTTTCCCTCCT |
| Crhr1 Forward | GGGCAGCCCGTGTGAATTATT |
| Crhr1 Reverse | ATGACGGCAATGTGGTAGTGC |
| Cplx3 Forward | TGGAGCGAGATGCACAGTTC |
| Cplx3 Reverse | GGGCAGACGGTATTTGTCTCT |
| Figf Forward | TTGAGCGATCATCCCGGTC |
| Figf Reverse | GCGTGAGTCCATACTGGCAAG |
| Nr4a2 Forward | GTGTTCAGGCGCAGTATGG |
| Nr4a2 Reverse | TGGCAGTAATTTCAGTGTTGGT |
| Pik3cg Forward | GCTCTTCGCCATCACACAAAC |
| Pik3cg Reverse | GGCATTCCTGTCATCAGCATC |
| Gpnmb Forward | TGCCAAGCGATTTCGTGATGT |
| Gpnmb Reverse | GCCACGTAATTGGTTGTGCTC |
| Ptger4 Forward | CCATTCCCGCAGTGATGTTCA |
| Ptger4 Reverse | TGCGCGACTTGCACAATACTA |
| P2rx7 Forward | GACAAACAAAGTCACCCGGAT |
| P2rx7 Reverse | CGCTCACCAAAGCAAAGCTAAT |
| Ppbp Forward | CTCAGACCTACATCGTCCTGC |
| Ppbp Reverse | GTGGCTATCACTTCCACATCAG |
| Postn Forward | CCTGCCCTTATATGCTCTGCT |
| Postn Reverse | AAACATGGTCAATAGGCATCACT |
| il-1b Forward | CTCACAAGCAGAGCACAAGC |
| il-1b Reverse | CAGTCCAGCCCATACTTTAGG |
| il-6 Forward | GCTGGTGACAACCACGGCCT |
| il-6 Reverse | AGCCTCCGACTTGTGAAGTGGT |
| inos Forward | CAAGCACCTTGGAAGAGGAG |
| inos Reverse | AAGGCCAAACACAGCATACC |
